# Investigating Cognitive Load in Energy Network Control Rooms: Recommendations for Future Designs

**DOI:** 10.3389/fpsyg.2022.812677

**Published:** 2022-03-28

**Authors:** Umair Afzal, Arnaud Prouzeau, Lee Lawrence, Tim Dwyer, Saikiranrao Bichinepally, Ariel Liebman, Sarah Goodwin

**Affiliations:** ^1^Faculty of Information Technology, Monash University, Melbourne, VIC, Australia; ^2^School of Engineering, Swinburne University of Technology, Melbourne, VIC, Australia; ^3^Inria and LaBRI, University of Bordeaux, CNRS, Bordeaux-INP, Bordeaux, France

**Keywords:** control room, cognitive load, eye tracking, energy market operators, situational awareness

## Abstract

This study analyzed and explored the cognitive load of Australian energy market operators managing one of the longest inter-connected electrical networks in the world. Each operator uses a workstation with seven screens in an active control room environment, with a large coordination screen to show information and enable collaboration between different control centers. Cognitive load was assessed during both training scenarios and regular control room operations via the integration of subjective and physiological measures. Eye-tracking glasses were also used to analyze the operators gaze behavior. Our results indicate that different events (normal or unexpected), different participants for the same session, and different periods of one session all have varying degrees of cognitive load. The system design was observed to be inefficient in some situations and to have an adverse affect on cognitive load. In critical situations for instance, operator collaboration was high and the coordination screen was used heavily when collaborating between two control centers, yet integration with the system could be improved. Eye tracking data analysis showed that the layout of applications across the seven screens was not optimal for many tasks. Improved layout strategies, potential combination of applications, redesigning of certain applications, and linked views are all recommended for further exploration in addition to improved integration of procedures and linking alarms to visual cues.

## 1. Introduction

Electrical networks and infrastructure are continuously monitored by control room operators to ensure the supply of secure, affordable and reliable electricity across the country. The Australia's National Electricity Market (NEM) is one of the longest inter-connected electricity networks in the world (approximately 40,000 km) and is comprised of transmission networks for five geographically connected regions of eastern and southeastern Australia. Electricity is generated, consumed, and traded across these regions. An incident on the network can quickly have a large impact as seen in September 2016 with the South Australian blackout. A line damaged by poor weather led, 90 s later, to the whole state going black. This initial event actually provoked a cascade of other line and power generator failures which illustrates the complex interconnection between the elements of the network that operators need to deal with and the speed at which failure can propagate. With the rapid transition to distributed energy resources (including renewable resources which need space and are location dependent), this operational and dispatching control room is in charge of monitoring a larger number of electricity generators, thus increasing the complexity of the situation operators need to monitor. The increased amount of data together with uncertainty and unknown factors induced by the growing presence of emerging technologies and smarter systems i.e., electric vehicles, energy storage devices, demand response programs, microgrids, and nanogrids (Liu et al., 2016), is of growing concern to network operators especially in critical situations, making failures potentially harder to identify and faster to propagate. The manner in which information is managed and processed by control room operators needs to be better understood to ensure these environments are designed suitably for empowering data-driven decisions.

To improve our understanding of such control room scenarios we propose an investigation of the cognitive load of the control room operators. Cognitive load, is defined as the amount of mental resources needed to perform a task (Sweller, 1988), we can better understand which task requires more mental resources, and thus, propose design improvements to the control interface that could reduce this load. In this study, we define cognitive load in accordance with the demands placed on an individual from undertaking and learning from a task, inclusive of mental workload, mental effort, and performance (Orru and Longo, 2019), in this case, how they impacted on the participants using Heart Rate Variability (HRV), eye tracking, and affect.

In this research article, we present a study in which we measure cognitive load of operators in a network control room during both normal operation and unexpected events. Cognitive load can be estimated using performance, physiological or subjective measures. In this study, we used electrocardiac activity as a primary measure of cognitive load, and used a subjective measure, the Workload Profile (WP) questionnaire (Tsang and Velazquez, 1996), to assess the domain of cognitive load that was most impacted by the task. We also tracked operators' activities by tracking their gaze using eye tracking glasses and assessed the operator emotional experience of each control session using the Positive And Negative Affect Schedule (PANAS) questionnaire (Watson et al., 1988). As this is an exploratory study, we dont have strong hypothesis regarding the cognitive load of the operators during the different situations and even between operators (as several factors can be involved like experience). Our goal is to observe how cognitive load evolve during a situation and identify what caused it and how the design of the operators' interface influenced it. However, the metric and the analysis methods were determined before the study.

Our results suggest that unexpected events led to higher cognitive load compared to normal situations. This increase is, of course, due to the operators having to deal with a more complex situation and the time pressure associated with it, but it is emphasized by other factors. First, operators tend to optimize the placement of their application in the workspace for normal operation, but when an unexpected event happens, operators' activities change and they have to switch to a more active mode. During this task they will perform more visual back-and-forth between applications, for instance, to get information or follow a procedure. Such long-distance visual transitions can increase cognitive load due to the visual search required and the time required to acquire a distant visual target. The procedures to follow when such events happen consist of word documents that operators need to manually find in repositories that contain hundreds of them. Operators have to manually search for them, and then find the correct location in the workspace to perform the required action. Finally, while communication is important during normal operation, it is scarce and mostly about communicating of information amongst operators. When unexpected events happen, the amount of communication between operators greatly increases to either discuss important decisions, or coordinate actions on the grid in real-time. In such situations, having situational awareness of what other operators are doing is crucial and not well supported by the current system. This leads to a lot of communications in which operators have to explicitly describe what they are currently doing. After discussing these factors, we propose, design recommendations to adapt the system to mitigate the issues.

The research article is structured as follows: After a review of the literature (Section 2), we outline our study procedure (Section 3), methodology and limitations. We then present our analysis (Section 4) and discuss the main insights (Section 5). These research concludes with recommendations (Section 6) for continued research and future control rooms design.

We contribute: (i) a study to measure the cognitive load of operators during normal operation and unexpected situations, (ii) a discussion of the factors that impact the cognitive load of operators, (iii) a set of recommendations for the design of future energy control systems.

## 2. Related Work

In this section we define and describe cognitive load. We note the difficulty of measuring these effects as we report prior work that has been done in this area, in particular, to understand cognitive load of operators in control room environments.

### 2.1. Cognitive Load Measures

Cognitive load refers to the amount of information processed by working memory in a given time and space (Dan and Reiner, 2017); the demands placed on an individual from undertaking and learning from a task. Here, mental load refers to the demands from the task itself, whereas mental effort is the step-by-step controlled or automatic processing an individual is engaged with. Indeed, both controlled and automatic processes can impact on task performance (Paas et al., 1994; Orru and Longo, 2019). Similarly, cognitive load theory differentiates three types of cognitive load: *intrinsic, germane*, and *extraneous* (Sweller et al., 1998; Dan and Reiner, 2017). Intrinsic load refers to the intrinsic and innate difficulty/complexity of understanding information or performing a task. It depicts the number of elements that are processed concurrently in the working memory for the construction of schema (Orru and Longo, 2019). Young et al. (2015) further informs that intrinsic cognitive load can not be changed by instructional interventions because it is intrinsic to the material being dealt with. Extraneous load refers to the extra demands placed on an individual by the way information is presented or instructed and is increased if ineffective methods are used. And hence, it can be altered by instructional interventions unlike intrinsic cognitive load (Young et al., 2015). Germane load depends on the effort put in by the individual to process information and construct a schema. After the reconceptualization of cognitive load theory, germane load is considered not an independent source of load but it is the function of those working memory resources concerning intrinsic load of a task (Orru and Longo, 2019). The intrinsic, extraneous, and germane loads are influenced by “unfamiliarity,” the way information and data are organized and displayed, and the effort required to process the information, respectively (Longo, 2018; Pawar et al., 2018). Cognitive load, after the reconceptulisation of cognitive load theory, is believed to be an additive consequence of the intrinsic and extraneous load, whereby if one is kept constant the other can be measured and vice versa. Whereas, measureability of germane load is not clear (Orru and Longo, 2019). In an optimal scenario there is a high level of familiarity with the event, data and information that is presented. The critical and relevant cues made are salient, and the cognitive effort needed to interpret this information is minimal. During higher levels of cognitive load scenarios, the operational performance of operators in power plants, cement factory, and traffic control centers declined. Higher levels of cognitive load over an extended period can cause chronic stress and mental fatigue (Fallahi et al., 2016a).

With the above notions covered, it is important to provide a basic level of some wider considerations around the terms and descriptions used above to prevent confusion. More specifically, we refer to notions that like aspects of cognitive load, the term mental workload has similarly been used to investigate and thus describe cognitive demands of a task too (Miyake, 2001). Thus, as previously mentioned, the difference between mental workload and cognitive load appears to pertain to the use of mental effort and related processes, which could extend more greatly onto the use of intrinsic or germane load, given cognitive load theory publications appear to more readily incorporate those terms (Orru and Longo, 2019) when compared to others that focus solely on task demands alone (Hancock et al., 2021). On the other hand, other literature has suggested that cognitive load and mental workload are the same construct (Naismith et al., 2019). In addition to this, mental workload has also been referred to as cognitive workload too, but these have been suggested to be the same construct (Orru and Longo, 2019). Given constraints, this article cannot properly elaborate on these points, but such discrepancies may be worth highlighting because they may explain mix and matching of the terms in the literature, and this impacts on this article. As mentioned, this study has adopted the definitions outlined in the paragraphs preceeding this one. Thus, we use cognitive load in this article to better reflect the holistic processes required of control room operators, especially during the contexts used in this study that require task demands, mental effort processing and schema construction and retrieval.

Measures of cognitive load can be divided into three classes: *performance-based measures, subjective measures*, and *physiological measures* (Eggemeier et al., 1991), in this work we will only use the two latter. Subjective measurements reflect the perceived cognitive load and the affective state of operators (Miller, 2001), and physiological measures focus more on how cognitive load is expressed in their body (e.g., heart rate). However, both have limitations and it is recommended to use them concurrently to allow for cross referencing between the subjective ratings and the physiological measures (Tsang and Vidulich, 2006).

Several questionnaires can be used to assess subjectively the cognitive load like the *Workload Profile* (WP) or the *NASA Task Load Index (TLX)*. The WP questionnaire is a multidimensional and subjective tool to assess Workload proposed by Tsang and Velazquez (1996). It asks participants to rate, on a scale from 0 (no demand) to 100 (maximum attention), the amount of attentional resources required on 8 workload dimensions: perceptual/central processing, response selection and execution, spatial processing, verbal processing, visual processing, auditory processing, manual output and speech output. The NASA task load index gives overall workload score based on a weighted average of ratings across six subscales (mental demands, physical demands, temporal demands, own performance, effort, and frustration) (NASA, 1986). Finally, other questionnaires are only unidimensional (e.g., the rating scale of mental effort; Ghanbary Sartang et al., 2016). Overall, evidence suggests that there is strong validity between all mentioned questionnaires (Longo, 2018), therefore the specific choice of measure can be argued to relate to the processes and time constraints involved in the specific task or study a researcher is conducting. Rubio et al. (2004) evaluated psychometric properties of three different subjective workload measures including Workload Profile (WP), NASA Task Load Index (TLX), and SWAT (Subjective workload assessment technique) and concluded that all the three subjective workload measures showed high convergent validity. For our study, we chose WP because it includes more relevant dimensions when assessing the cognitive load in control room environment due to the number of auditory and visual stimuli, for instance. In the control room environment, for instance, different operators may have to move around and verbally communicate with each other and hence the dimensions of verbal processing, auditory processing, and speech output are relevant. Also, during different normal and unexpected situations operators may need to perceive the problem, spatially process the data spread across multiple screens, and then select responses and execute.

Multiple resource theory forms the basis of the WP. The WP is built of principles associated with mental workload, but is intended to be used for cognitive load in this study. To be brief, multiple resource theory separates mental processing into *stage, modality, code*, and *responses*. Stage differentiates the mental resources that are required for initial perceptual and cognitive activities that are required for choosing and eventually executing responses. Modality differentiates processing between auditory and visual sources, and similarly both *codes* and *responses* can be categorized into spatial and verbal processes. It should be noted that two tasks that involve the same dimension (i.e., *stage, modality, code*), are likely going to compete for limited resources, whereas tasks that are unrelated are less likely to be affected (Wickens, 2008). Additionally, if two concurrent tasks exist with one requiring either perceptual or cognitive processing and the other more responses, then the change in difficulty in one is less likely to affect the other because the stages can utilize different mental resources (Wickens, 2008). As a more clearly defined example, Parkes and Coleman (1990) demonstrated that route guidance was best delivered auditorily rather than visually when subjects were driving a simulated vehicle at the same time; driving already involving significant visual-spatial load. Hence, cross-modal demands have an advantage over intra-modal demands, and this seems similar for codes too (Wickens, 1980).

Tao et al. (2019) identified 78 physiological measures which have been considered and tested to be effective agents of cognitive load. These measures were distributed across a variety of physiological processes; the most common categories being cardiovascular, EEG, and eye movement measures. Across the 91 studies reviewed in this survey, 72% of them suggest that the physiological measures have shown promise in tackling the problem of cognitive load modeling, but their validity and wide applicability still have to be demonstrated across experiments.

Heart Rate Variability is the variation of the length of heart beat intervals (Malik and Camm, 1990). HRV is shown to have an inverse relationship with cognitive load (Myrtek et al., 1994; Fallahi et al., 2016b). Such measure has been successfully used in studies involving participants with chronic mental stress (Kim et al., 2008), and also with ship operators (Wulvik et al., 2020), where lower HRV is associated with greater physiological arousal that greater cognitive load has shown to induce (Wulvik et al., 2020). HRV measure was preferred over EEG measure in our study because acquiring EEG signals would mean attaching electrodes onto the scalp of operators which would be more intrusive as well as it is more susceptible to noise due to the movement of participants.

Zagermann et al. (2016) concluded that eye tracking can be valuable agent to measure and analyse the cognitive load in the context of Human-Computer Interaction (HCI) and visual computing. Eye tracking has been linked to cognitive load, when using microsaccades, and measuring the mean change of rate and magnitude (Krejtz et al., 2018). High cognitive load is associated with a lot of quick eye movements, taking on board numerous pieces of information together, which visual scanning facilitates (Krejtz et al., 2018). Bhavsar et al. (2017) explored the association between eye gaze behavior and the cognitive steps involved in orientation, diagnosis, and execution phases of control room operations in the process industry. In this environment the majority of accidents (70%) are caused by human error. In the study, participants who successfully managed disturbances had significantly lower values for gaze transitions and lesser fixation dispersion in the execution phase. By contrast, both successful and unsuccessful participants had similar levels of fixation dispersion and gaze distribution recorded during normal operations, when no abnormalities were observed. In conclusion, after the abnormality has been flagged, successful participants fixated on the relevant variables and manipulated them to manage the abnormality and recorded very low dispersion in fixation. In our study we measured the gaze and eye movements of the participants during each session to understand which screen of their workstation they were looking at, how much they moved their gaze between screens and at what frequency, and what applications they were using to investigate the cognitive load of the operators.

### 2.2. Cognitive Load in Control Rooms

In emergency centered scenarios, there is a potential issue of information presentation inefficiencies and sub optimal situational awareness which can potentially lead to overload of cognitive demand. To deal with such situations operators deploy strategies to circumvent information complexity to handle the situation. The strategies identified and observed are omission, reducing precision, filtering, extrapolation, similarity matching, random trial and error, escape, and queuing (Hollnagel and Woods, 2005). The strategies can lead to failure of the system and while lowering cognitive load may also deteriorate the necessary situational awareness.

Both excessive information and lower levels of information than required prolonged the time duration of diagnosing the fault and clearing the alarms (Dadashi et al., 2016). Interestingly, the alarm episodes with “high information” took longer than those with “low information”. When observing Nuclear Power Plant operators, it was noted that more than 50% of alarms were redundant and were actually decreasing the situational awareness of the operators (Mumaw et al., 2000). Studies also indicate that a significant number of operators could not react appropriately to the critical alarms and demonstrated “inattentional deafness” due to their limited attentional resources when performing critical tasks (Giraudet et al., 2014).

One way to limit cognitive load is to use cues. Cues are referred to the specialized associations between specific situations and the environmental features or objects (Brouwers et al., 2016). The association of cue utilization with cognitive resource consumption was observed for DNSP (Distribution Network Service Providers) control room operators (Sturman et al., 2019). Operators with higher cue utilization showed a lower cognitive load. It was demonstrated and supported by the observation that operators with higher cue utilization showed lesser levels of cerebral oxygenation rise in the prefrontal cortex from baseline, indicating the consumption of cognitive resources at a slower rate (Sturman et al., 2019). The cognitive resources are limited and a system should be designed to conserve them as much as possible for they might be needed, shall a critical situation arise. Dehais et al. (2014) concluded that earlier exposure to a critical event enhanced subsequent alarm detection for an event. In this research article, we propose guidelines to limit cognitive load in the specific context of network control rooms.

### 2.3. Guidelines for Control Room System Design

Liu et al. (2016) observed that the conventional energy management system does not have the appropriate functions to provide for adequate situational awareness, as there is a lack of understanding of the dispatcher's thought process. Endsley (1995) emphasized that the interface design should be situational awareness orientated, rather than simply technically orientated, so operators can quickly and efficiently perceive, comprehend, and predict the situation and make more informed decisions.

According to Giri et al. (2012), the future of grid management is moving away from the current “observe and control” reactive paradigm toward a more integrated proactive paradigm; one that does not just indicate problems but proposes “corrective actions.” For instance, a new energy management system was deployed in RTE (Rseau de Transport dElectricit, the French Transmission System Operator) that includes a predictive model that anticipates conditions for the next 48 h (Astic et al., 2018).

Whilst automation technology and the advancement in power electronics has greatly enhanced reliability of electrical equipment, humans are still essential to the operation and decision making process. Evidence from problem events indicates that many are partially or solely due to human error. Analysis of the North American blackout of 2003, for example, identified that one of the reasons for an eventual cascading failure and blackout was the lack of monitoring of the state of the grid (Muir and Lopatto, 2004). Automation may lead to lower cognitive load but may also deteriorate situational awareness of the operator and hence, ideally, an adaptive automation system design would keep the human operator “in the loop” (Tsang and Vidulich, 2006). The ideal system design would support reduced cognitive load, but increased situational awareness (Endsley, 1995).

## 3. Study

We present a study in which we measure the cognitive load of operators in an electrical network control room. More specifically, we aim to identify cognitively heavy tasks and their causes (e.g., long procedures or information scattered in the interface). As operator tasks are complex and mostly collaborative, they are hard to reproduce in laboratory settings without simplifying the task or adapting it to the individual context. Both these characteristics are the main contributors of cognitive load, and keeping them at a level that is as ecologically valid as possible is thus very important. For this reason, we decided to perform this study “in the wild” during real-time operations in a control room.

While doing a study “in the wild” provides a more ecological environment, it also makes it difficult to control the different situations that operators will face. In work exploring Road Traffic Control and Nuclear Power Plant scenarios (Prouzeau, 2017), Prouzeau defined two types of situations that operators experience while monitoring a system: (1) *Normal Situations*, in which the system operates normally and each operator has precise tasks, which mainly consist of monitoring parts of the system. Operators have to follow precise procedures that are designed to limit the need for intense collaboration; (2) *Exceptional Situations*, in which an unusual event provokes an important disturbance in the monitored system. The operators need to perform additional tasks that generally require a global view of the state of the system and thus to collaborate with the other operators. All of this can substantially increase the cognitive load.

Exceptional situations are thus the ones that are more likely to provoke cognitive fatigue. However, they are also very rare and the chance to encounter one during our study during real-time operation was substantially low. To this end, we will in addition perform the same study during training sessions in which operators are only facing exceptional situations. While this is of course not the same as encountering such situations during real-time operations, it provides us a good proxy to assess cognitive load in such situations (Rao et al., 2020).

As defined by Tukey (1977), the study we are doing here is observational and exploratory. We do not aim at testing strong hypothesis but rather at exploring how cognitive load evolve during a specific situation and after an unexpected event and try to understand better how the system (and its interface) have an impact on this evolution. The results of this study suggest recommendations for the design of control room systems but also new research directions to develop (and in which hypothesis testing methods could be used) (Wicherts et al., 2016).

### 3.1. Context

The study was performed in the Australian Energy Market Operator (AEMO) control room in Brisbane which is in charge of the control of the network in the states of Queensland, Victoria, and Tasmania. Another control room located near Sydney is in charge of the network of New South Wales and South Australia. As the networks in all states are deeply interconnected, the two control rooms are in constant communication using videoconferencing.

In each control room there are two operators, one in charge of each state (Victoria and Tasmania are controlled by the same operator because of the high inter-dependency between their network). In addition, there is an operator, the shift manager, who is in charge of coordination between the different states. The shift manager is either located in the control room in Brisbane or in Sydney.

**Figure 1:**
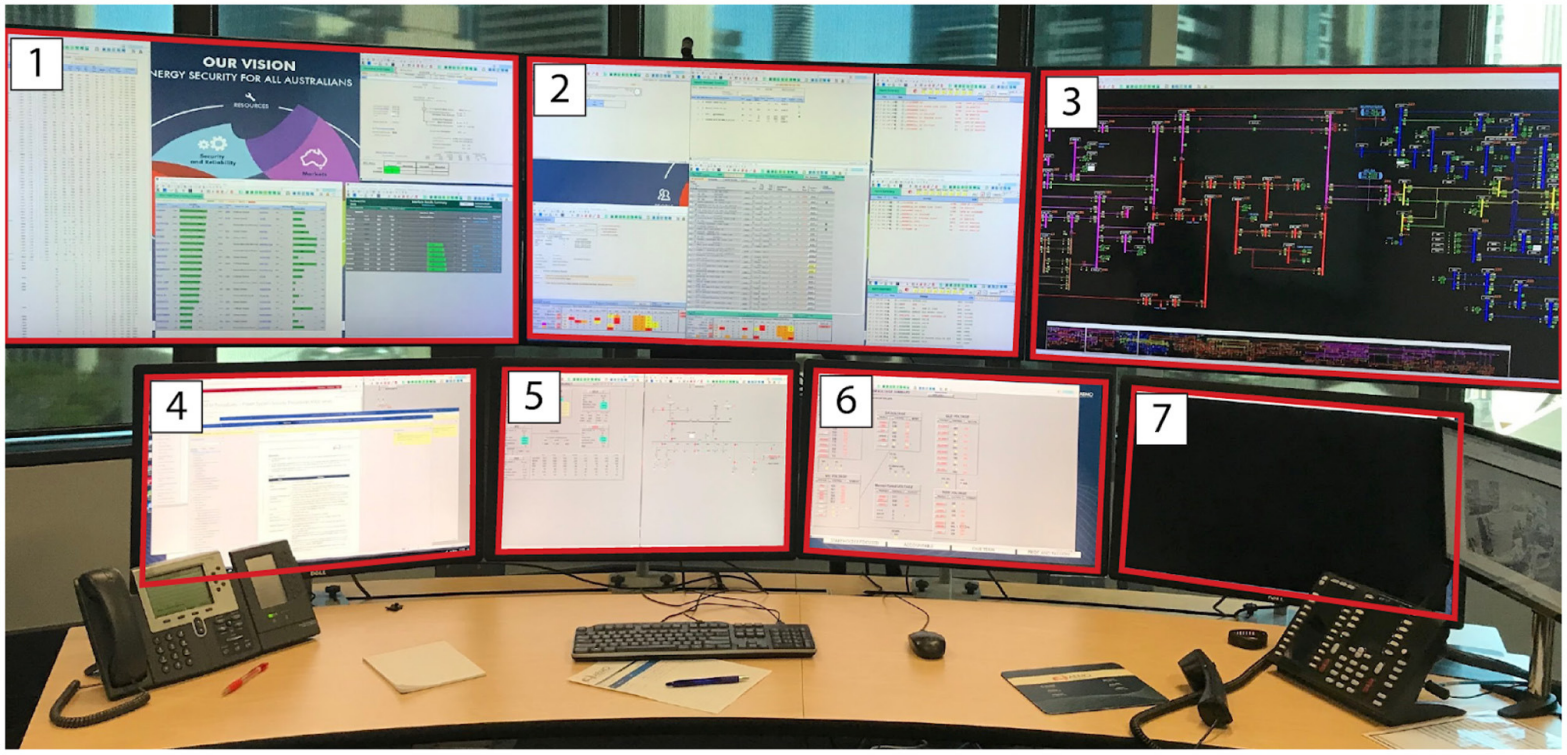
A typical workstation of an operator at the AEMO Brisbane control center, with 7 screens, a mouse, a keyboard, and two phones. The height of the entire desk can be changed by the operator.

Each operator has their own workstation composed of 7 screens (see Figure 1). There is a large wall-display that constantly shows contextual information that could be useful to operators (e.g., News channel, weather forecast, etc), a table for meetings, and a vertical interactive surface that is used only for the video-call with the other control room (constantly on). The training is done in a room that is a reproduction of the real control room, with the exception that there is only one large display that is used for both showing contextual information and the video-call.

Each operator has to go through one training session every year. Training sessions are organized in both centers at the same time (to reproduce realistic conditions), and 2–3 operators participate in each center. A training session lasts 2 days and is a mix between classes on specific topics and practical sessions in which operators have to deal with exceptional situations. These situations are based on real-life disturbances including weather events (including bushfires, storms, etc.), IT failure, and cyberattacks.

### 3.2. Procedure

The study took place over 4 days. During the first 2 days, we collected measurements for two operators as they completed three training situations (Called in this research article: Training 1, Training 2, and Training 3). Training 1 and Training 3 lasted around 120 min and were thus divided into two sessions to allow for operators to have a break. Training 2 lasted 60 min and was carried out without a break. Each training session is divided into different phases corresponding to different events happening in the simulation, these phases were defined by the training staff and we used them in our analysis to observe the evolution of cognitive load during the session.

In the final 2 days, we measured the cognitive load of two operators in the operational control room twice a day (called in this research article Real-Time Operation 1 and Real-Time Operation 2, where each session was approximately 60 min once in the morning and once in the afternoon). The first measurement, called handover, was at the start of the operators' shift. Incoming operators get briefed by the operators finishing their shifts about the state of the system in order to acquire necessary situational awareness of the grid. The second measurement, called normal operation, occurred in the afternoon as operators performed their regular duties. Overall, we recorded a total of 16 sessions: 2 operators × (5 training sessions + 4 regular operation sessions) - (see Table 1).

**Table 1:** Timetables of the different sessions (both the Training and the Normal Operation) for P1, P2, and P3.

Day 1	Training 1	2 Sessions	P1 and P2
Day 2	Training 2	1 Session	P1 and P2
	Training 3	2 Sessions	P1 and P2
Day 3	Handover 1	1 Session	P1 and P3
	Normal Operation 1	1 Session	P1 and P3
Day 4	Handover 2	1 Session	P1 and P3
	Normal Operation 2	1 Session	P1 and P3

**Figure 2:**
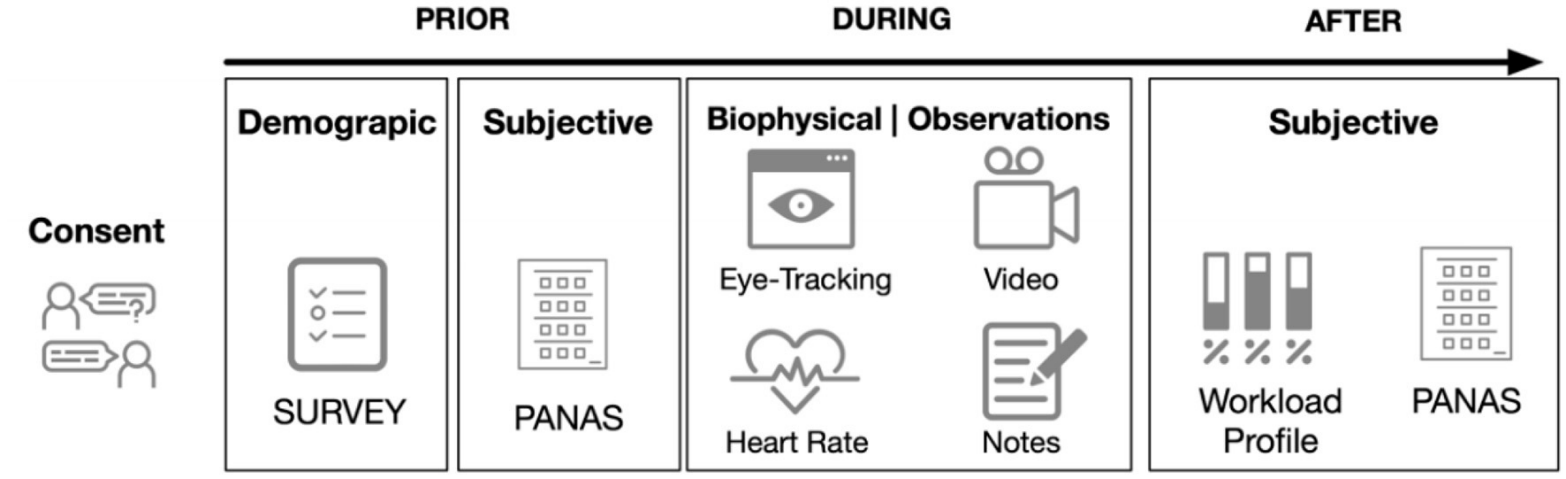
The typical procedure for one session of the study prior, during and after the recording.

The procedure for a session is shown in Figure 2. At the beginning of each session, the participants answered a demographic questionnaire a blank questionnaire given to the participants is provided as Supplementary Material) and a subjective questionnaire (more details regarding the questionnaire are given in the next section). The participants were then equipped with an electrocardiogram (ECG), with the electrodes attached to both wrists of the participants. Finally, they were equipped with eye tracking glasses, which required calibration.

During the session, three experimenters were always in the room. One taking notes and managing the video. The other two were in charge of one operator each and monitored both the ECG and Eye Tracking data, in order to make sure that the data is well recorded. However, as our study happened during real-time operation or official training, in case of issue with the data, it had to be fixed from the computer; it was not possible to re-calibrate or restart a device in the middle of the session.

At the end of each session, all devices were switched off, disconnected, and put on charge. Participants were asked to complete two subjective questionnaires. Electrodes were not changed between the sessions of the same day but disposed of after the final sessions of a day.

### 3.3. Measuring Cognitive Load

In this study, we decided to use a physiological measure, the Heart Rate Variability, and a subjective measure, the Workload Profile. In addition to cognitive load, we took another measure to understand the emotional states of the operator before and after the session using the PANAS questionnaire (Watson et al., 1988), and what activities they were doing (e.g., what application they were looking at) using eye tracking glasses and monitor observations.

#### 3.3.1. Heart Rate Variability (HRV)

To calculate HRV, we measured the heart rate of the participant during the session using an electrocardiogram device (Shimmer3 ECG^1^) which leverages MSP430 microcontroller (24MHz, MSP430 CPU) and was placed in a bipolar topology (wrist to wrist). The data were sent in real-time to a computer in the same room via Bluetooth Radio RN - 42 at a frequency of 512 Hz. After the study, the data were manually cleaned, and we used the Root Mean Square of Successive Differences (RMSSD) to get the HRV (Qin et al., 2017).

We decided to use RMSSD over other measures of HRV (SDNN, pNN50, NN50) because it has been used in ultra short (10 s), through to long duration recordings (24 h) (Shaffer and Ginsberg, 2017). This versatility is the predominant reason why RMSSD was chosen in our study over SDNN and pNN50, because the latter two require 60 s through 2 min. RMSSD more greatly reflects parasympathetic activity when compared to SDNN (SDNN is influenced by both parasympathetic and sympathetic influences), which is noteworthy considering participants were greatly considered as being “at rest” in this predominant non-emotion induction, cognitive load study. Nevertheless, all of RMSSD, SDNN, and pNN50 are highly correlated. NN50 or pNN50 also investigate parasympathetic activity, focusing on the number of adjacent beat-to-beat intervals that differ by more than 50 ms. Thus, NN50/pNN50 tends to be more of a “categorical” classification of heart beats rather than the more “continuous” or “metric” aggregation that is used with RMSSD and SDNN, and we wanted to use a more ‘continuous’ approach in case few occurrences of 50 ms differences occurred.

In the case of real-time operation, the HRV was calculated for the entire session. For the training sessions, it was calculated for each phase. We used the wrist-to-wrist configuration to limit the intrusiveness of the procedure (best practice would have been to stick electrodes on the chest of participants). However, this configuration led to more noise in the data due to the electrode rubbing on the desk of the workstation. While most of the noise could be removed when cleaning the data, a few sections of the data had to be discarded.

#### 3.3.2. Workload Profile (WP)

The WP questionnaire has to be filled retrospectively, in our case, at the end of each session. In the case of real-time operation, participants gave one rating for each dimension for the entire session from zero to hundred. The dimensions include perceptual/central processing, response selection and execution, spatial processing, verbal processing, visual processing, auditory processing, manual output and speech output. For the training sessions, they gave one rating for each dimension for each phase. To calculate a total Workload Profile score, we averaged the score of each dimension (See equation 1, with *X*_*D*_ being the score for the dimension *D*). In the case one user did not fill a dimension, we did not count it in the average as explained by Tsang and Velazquez (1996). A blank table given to the participants is provided as Supplementary Material. As per the instructions, participants are asked to read the definitions of each of the domains (see in the attached Supplementary Material), and then respond under each column in accordance to how much the task involved of that particular domain. Participants answer in the boxes for each domain in accordance to the effort required of them in the task they just did. To do this, they use a 0 to 100 value (which can be thought of as 0–100%)

(1) $$WP=\frac{\sum_{D\in DimensionsFilled}X_{D}}{\#DimensionFilled}$$

As a note, Tsang and Velazquez (1996) states that it is possible for a user to not give a score for a dimension of the WP if they fell the task did not involve this specific dimension. The situation in which two participants did not fill the same dimension of the WP is not problematic in our study because we did not compare their score for one session but the evolution of the WP between the different phases in one session for each operator. The reason for this is that the two operators could do different tasks, as they have different roles.

#### 3.3.3. Positive and Negative Affect Schedule (PANAS)

The PANAS is a questionnaire to measure positive and negative emotions of the participants developed by Watson et al. (1988). They have to rate on a scale from 1 (not at all) to 5 (very much) their feelings in terms of 10 positive and 10 negative emotions. The scores for each affect are then summed to get a score for the positive affect and a score for the negative one. In this study, we were interested in the impact of the session on the participants' affect, so they completed the questionnaire both at the beginning and at the end of the session to obtain a difference score. A blank questionnaire given to the participants is provided as Supplementary Material.

#### 3.3.4. Eye Tracking

Because of the size of the individual workspace (around 3 m long and 1 m high), we could not use a regular screen eye tracker. We therefore used the Tobii Pro glasses 2 wearable eye tracker that samples at 50 Hz and have an accuracy of around 0.62^2^. In addition to the gaze position, the glasses also record a video feed from the perspective of the wearer. A picture of the workspace was taken before and after each session to then map the gaze position to it using the Tobii Pro software. While most noise in the data was cleaned by the software, we also performed a manual cleaning and discarded data that seemed corrupted due to exterior reasons (e.g., hair falling in front of the glasses or issues due to the use of prescription glasses). The data were sent to a computer using WiFi during the study. Due to technical constraints, only one operator could wear the glasses at each session (P2 and P3).

#### 3.3.5. Observation

All the sessions were video recorded with audio. In addition, one experimenter took notes of interesting events that happened with the timing, and took pictures of the applications used in the screen in such cases. These were used to understand better the context of the session and identify the timing of the phases in training, important events in general, and the different applications used by the operators.

### 3.4. Participants

At any one session there were two participants who were recorded in parallel for approximately 60 min at a time. In total, three operators (P1–P3) participated in the study over the 4 days, where eight sessions took place. Each participant had varying degrees of control room experience: less than 1 year (P1), 15 years (P2), and nearly 30 years (P3). Prior to each session the participant signed a new consent form approved by Monash University's ethics committee. P1 and P2 participated in training scenarios on days 1 and 2 wherein a variety of virtually simulated disturbances were introduced and they had to manage the complex situations along with the team in another control center in a different state. Then, P1 and P3 participated in normal control room operations on days 3 and 4.

The number of participants for this study were small (i.e., three) and hence the ability to compare the performances and expertise of operators is limited. Two participants had substantially more experience than the third but the two were never doing the same tasks. During training scenarios and the control room operations, at any one time two operators were performing the duties of the management of the NEM along with one shift manager.

## 4. Results

The data from the eye-tracker and the ECG were first cleaned and preprocessed and then synchronized. Using the video and the notes from the session, it was possible to find the timestamps of the beginning and end of the different phases (for the training scenarios). The eye-tracker data was mapped to a photo of each operators workstation—a snapshot taken after each recording. For this project, we defined 7 Areas Of Interest (AOI) corresponding to the 7 screens of the operators workstation (see Figure 1). We then calculated and mapped the amount of time each participant spent gazing within or moving between the AOIs during each phase of each session. Similarly, the HRV was computed for each phase. Finally, we calculated the positive and negative affect score from PANAS and calculated the WP, which reports the average score the participants gave to each criterion. In the remainder of this section, we will briefly explain the context of each session and our findings regarding their cognitive load and activities. The Tables 2–4 shows the results to the PANAS questionnaire for each session for each participant. Then, individual figures show the results of the other measures for each session. In each figure, the top-left chart shows the evolution of the WP and HRV during the session for both participants (the axis of the HRV is inverted). The top-right chart shows the evolution of each criteria of the WP during the session for both participants. In some sessions, participants judged that a criteria of the WP was not relevant and did not indicate any value. In such case, we chose to not plot it in that case instead of considering it as 0. The bottom chart displays eye tracking data for P2 for each phase: at the top is the schematic representation of the operator's workspace with a heatmap showing the amount of time the operator spent focusing on each screen; the bottom graph indicates the amount of transition between each of the screens with the thickness of the lines.

**Table 2:** Result table of the PANAS questionnaire for P1.

	**P1**			
	**Positive**		**Negative**	
	**Before**	**After**	**Before**	**After**
Training 1-Session 1	42	44	16	17
Training 1-Session 2	37	40	12	12
Training 2	-	-	-	-
Training 3-Session 1	36	41	12	15
Training 3-Session 27	35	45	10	16
Real-Time Operation 1-Handover 1	25	31	11	12
Real-Time Operation 1-Handover 2	31	26	11	10
Real-Time Operation 2-Normal Operation 1	-	-	-	-
Real-Time Operation 2-Normal Operation 2	25	28	10	10

*For each session, the score of both participants' positive and negative affects is calculated. The color indicates the evolution of the participant's mood during the session: Green if it improves (score increase for the positive affect and decrease for the negative one), yellow if it stays the same, and red if it deteriorates (score decrease for the positive affect and increase for the negative one)*.

**Table 3:** Result table of the PANAS questionnaire for P2.

	**P2**			
	**Positive**		**Negative**	
	**Before**	**After**	**Before**	**After**
Training 1-Session 1	37	38	10	10
Training 1-Session 2	37	32	10	10
Training 2	37	36	10	10
Training 3-Session 1	31	39	10	10
Training 3-Session 2	40	31	10	13

*For each session, the score of both participants' positive and negative affects is calculated. The color indicates the evolution of the participant's mood during the session: Green if it improves (score increase for the positive affect and decrease for the negative one), yellow if it stays the same, and red if it deteriorates (score decrease for the positive affect and increase for the negative one)*.

**Table 4:** Result table of the PANAS questionnaire for P3.

	**P3**			
	**Positive**		**Negative**	
	**Before**	**After**	**Before**	**After**
Real-Time Operation 1-Handover 1	37	40	12	10
Real-Time Operation 1-Handover 2	37	41	13	12
Real-Time Operation 2-Normal Operation 1	25	25	10	10
Real-Time Operation 2-Normal Operation 2	40	41	12	11

*For each session, the score of both participants' positive and negative affects is calculated. The color indicates the evolution of the participant's mood during the session: Green if it improves (score increase for the positive affect and decrease for the negative one), yellow if it stays the same, and red if it deteriorates (score decrease for the positive affect and increase for the negative one)*.

### 4.1. Training 1: Separation of Two Networks (P1 and P2)

#### 4.1.1. Context

**Figure 3:**
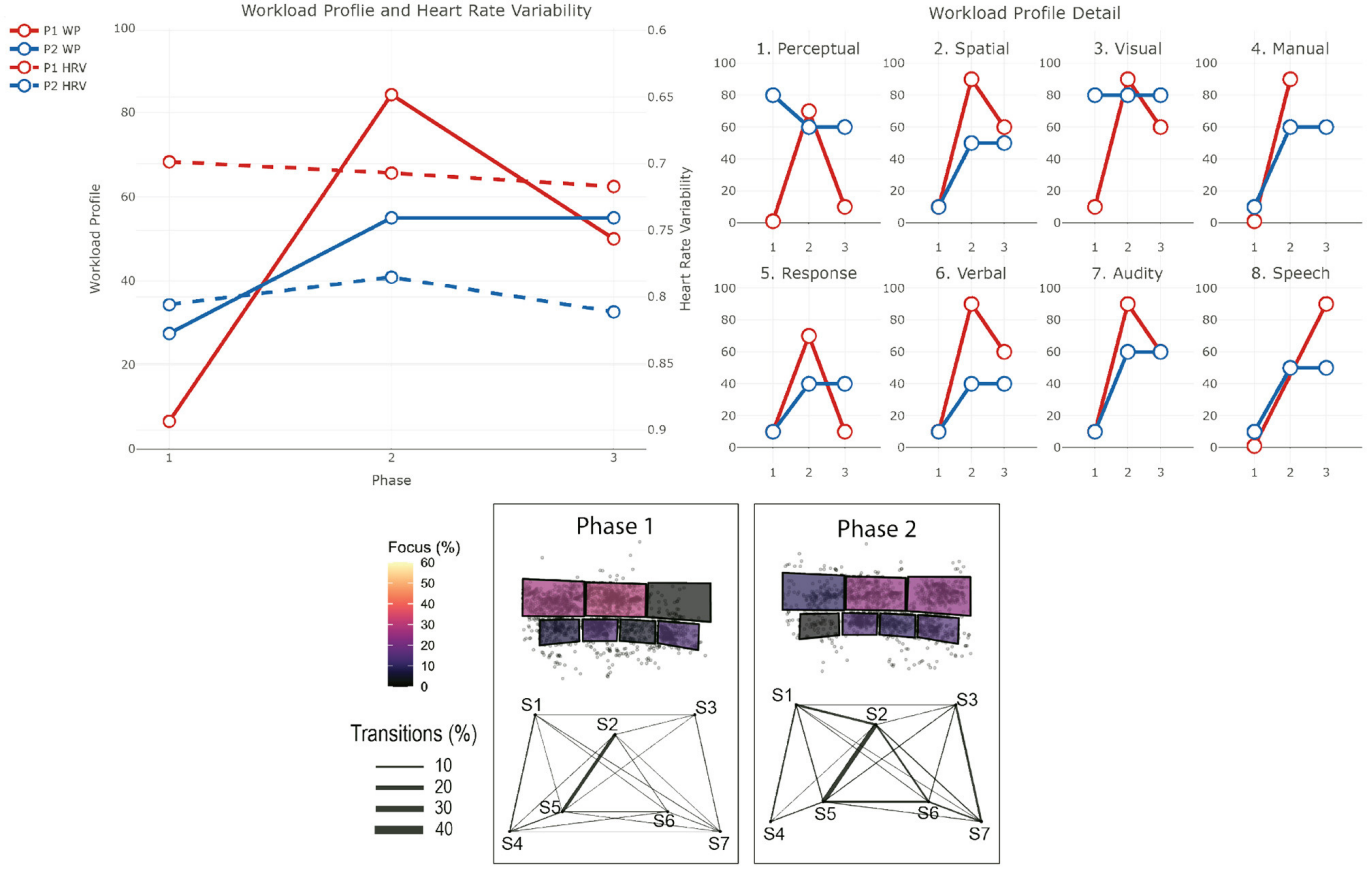
Results for the first session of training 1. The top-left chart shows the evolution of the WP and HRV during the session for both participants (the axis of the HRV is inverted). The top-right chart shows the evolution of each criteria of the WP during the session for both participants. The bottom chart displays eye tracking data for P2 for each phase: at the top is the schematic representation of the operator's workspace with a heatmap showing the amount of time the operator spent focusing on each screen; the bottom graph indicates the amount of transition between each of the screens with the thickness of the lines. The same organization is used in the following figures.

**Figure 4:**
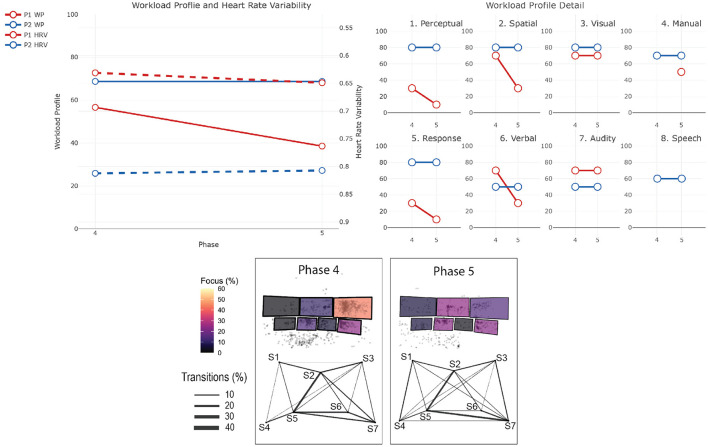
Results for the second session of training 1. The top-left chart shows the evolution of the WP and HRV during the session for both participants (the axis of the HRV is inverted). The top-right chart shows the evolution of each criteria of the WP during the session for both participants. The bottom chart displays eye tracking data for P2 for each phase: at the top is the schematic representation of the operator's workspace with a heatmap showing the amount of time the operator spent focusing on each screen; the bottom graph indicates the amount of transition between each of the screens with the thickness of the lines. The same organization is used in the following figures.

This training is divided into two sessions. In this training, the operators face a series of non-related incidents at the border of Victoria and New South Wales which ultimately leads to the separation of the two networks. Such separation is problematic as it forces each network to rely only on their generation capabilities and thus be more sensitive to incidents. The scenario starts with a “Work as usual” phase (*Phase 1*), in which operators are asked to perform routine tasks and get awareness of the current network situation. Then, operators receive a notice that a line in Victoria has to be taken out for maintenance reasons (*Phase 2*). This is also a routine operation and helps operators getting a good overview of the situation. When the operation is over, the first incident starts with two lines failing on the west part of the border between the two states (*Phase 3*). While these lines supply only a few places, and thus are not vital for the connection of the two networks, they provide electricity to a mine and operators are pressed to restore the power as miners are stuck underground. The first session ends with the resolution of this situation. The second session starts with a call to inform operators that sparks have been seen on one of the main lines that connect both networks, and thus needs to be taken out immediately (*Phase 4*). This forces operators to reconfigure the network as there is only one connection left between the two networks. Finally, a bushfire, exacerbated by strong winds, threatens this last connection and operators have to consider a potential separation and reconfigure the networks accordingly (*Phase 5*). The two lines that make up that last connection finally fail which provokes the separation event. P1 and P2 participated in this session. In addition, a manager in the same room and 2 operators in a control room in another state participated in this training. Figure 3 shows the results for the first session and Figure 4 shows them for the second session.

#### 4.1.2. P1 - Session 1

*Workload Profile*

In the initial phase of this session, P1's workload is low (WP = 7), but it increased abruptly with the short notice outage (WP = 84), with an increase in all the dimensions. In the last part of the session, when the two lines failed, their workload decreased to medium level workload (WP = 50). Interestingly, while we can see a decrease in all dimensions of the WP, the one related to the *stage of processing* (*Perceptual* and *Response*) decreased more than the others, and to the same level as in the beginning of the scenario. Here, we can suggest that P1 is mostly observing the others handling this last situation.

*Heart Rate Variability*

The HRV suggests that their stress level decreased during the entire session (HRV = 0.69 at the beginning and HRV = 0.72 at the end), which could be explained by the fact that they are still an operator in training, so they may feel anxiety before the session, and then they mostly observed at the end.

*PANAS*

The PANAS questionnaire confirm a potential feeling of anxiety as P1 indicated being moderately nervous and extremely jittery before the session. The session was seen as overall positive (positive score of the PANAS increased by 2), even when P1 felt more nervous at the end of the session.

#### 4.1.3. P2 - Session 1

The eye tracking glasses got disconnected from the computer at the end of phase 2 and we could not fix it on the moment, we thus lack eye tracking data for phase 3.

*Workload Profile*

P2's workload is low at the beginning of the session (WP = 28). This load is mainly due to *Perceptual* processing, which suggests that they is mainly monitoring and gathering awareness of the current situation, and to the processing of *Visual* input (most probably information on the screens, it will stay stable during the entire session). Similarly to P1, their workload increased with the short notice outage (WP = 55). The decrease of *Perceptual* processing and the increase of *Response* processing suggest a switch from monitoring to action in order to manage the outage. The management of this event includes more processing of *Auditory* input (e.g., Phone conversation with people on site) and both *Manual* (e.g., typing, clicking) and *Speech* responses (e.g., conversations). The workload is stable in the last part of the scenario.

*Heart Rate Variability*

The HRV, and thus the stress level, correlated with the WP between the first two phases as HRV = 0.80 at the beginning and HRV = 0.78 at the second phase. In the last phase, the HRV suggests a decrease of P2's stress level (HRV = 0.81).

*Screen Usage*

During this event, P2's focus was mostly on screen 1 (over 35%) and screen 2 (over 25%). There is a lot of back and forth between Screen 1 and 4, and Screen 2 and 5. When looking at the time spent on each screen, P2 did not spend that much time on Screens 4 and 5 which could mean that the visit was just to gather information on these screens.

*PANAS*

Overall, the session was positive for P2 as they reported feeling more excited at the end. P2 did not report any negative affect either before or after the session.

#### 4.1.4. P1 - Session 2

*Workload Profile*

The second session started strong with one line needing to be taken out. P1's workload is at mid-level (WP = 56). But surprisingly, the workload decreased with the last event of the scenario which provoked the separation of the two states' networks (WP = 39). We can see a decrease in *Perceptual, Response, Spatial*, and *Verbal* processing, which could mean that, as in session 1, P1 is mostly observing in the last phase.

*Heart Rate Variability*

This decrease of workload is correlated with a decrease of P1 stress level (HRV = 0.63 at the beginning and HRV = 0.65 at the end).

*PANAS*

P1 felt more Alert, Determined, Attentive and Active, which could suggest that they are not cognitively overloaded at the end of the simulation.

#### 4.1.5. P2 - Session 2

*Workload Profile*

P2's workload is high at the beginning of this session (WP = 69) and stayed stable throughout the session. With a *Perceptual* and *Response* processing rated at 80, we can see that they are highly involved in the response to both events of the session.

*Heart Rate Variability*

Their stress level is stable during the session (HRV = 0.81).

*Screen Usage*

Eye tracking data showed that P2's focus was mostly on screen 7 during the entire session, and spent around 20% of the time on screen 3 during the separation. On screen 7, P2 mostly used the *constraints management* application whereby the operator creates and invokes constraint equations according to the state of the grid networks.

*PANAS*

While P2 did not report any negative affect both before and after the session, we can see a decrease of positive affect; P2 felt less Alert, Determined, Attentive and Active, which could indicate P2 was impacted negatively by the cognitive demands placed on them; perhaps being an early indication of or consequence of being cognitively overloaded at the end of the simulation.

#### 4.1.6. Summary

In the first session, both operators agreed that the workload increased between Phase 1 and Phase 2, which can be explained by the fact that they had to deal with a short notice outage. Stress decreased during Phase 3, which could be a sign that this Phase is less cognitively demanding than the previous ones. This is reflected by P1 in the reduced workload profile in Phase 3 compared to Phase 2. P2s rating of the workload is similar in both phases. This is surprising as operators faced an unexpected event in Phase 3, which required both more actions and discussions. The reduction of the cognitive load between these two phases should be analyzed more deeply, but two possible reasons can explain this. First, more breaks took place during Phase 3 in order to analyse and discuss operators decisions during which the simulation was paused. This can again make the situation overall less stressful and limit time pressure. Second, as they already faced an unexpected event in Phase 2, operators are more prepared in Phase 3.

In the second session, for both operators, the subjective workload followed the pattern seen in HRV. P1 felt workload decreased, while on the contrary P2 felt it was high and stable. Additionally, the PANAS result shows that P2 felt less Alert, Determined, Attentive and Active, which could indicate P2 was impacted negatively by the cognitive demands placed on them; perhaps being an early indication of or consequence of being cognitively overloaded at the end of the simulation. This difference can be explained by several reasons. In general, their role was different, it is possible that P2s role was more cognitively intense in Phase 2 than P1s. By having a look at the screen usage, we can see that P2 spent a lot of time focusing on Screen 7 which contains the Constraint Management application. Interviews and observations later in the study suggest that this application is particularly demanding to use and can be slow and unresponsive, when needed urgently to put in market constraints in case of credible contingency events and the lack of confirmation pop-ups make it susceptible to erroneous editing, therefore extra attention is needed. In addition, the frequency of transitions between Screens 5 and 7 suggest that the placement of the application is not optimal for this task and it could explain an increased workload.

### 4.2. Training 2: Cyber Attack (P1 and P2)

#### 4.2.1. Context

**Figure 5:**
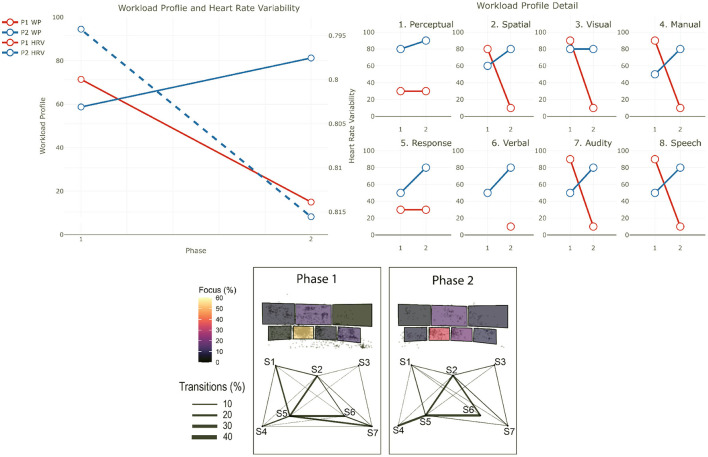
Results for training 2. The top-left chart shows the evolution of the WP and HRV during the session for both participants (the axis of the HRV is inverted). The top-right chart shows the evolution of each criteria of the WP during the session for both participants. The bottom chart displays eye tracking data for P2 for each phase: at the top is the schematic representation of the operator's workspace with a heatmap showing the amount of time the operator spent focusing on each screen; the bottom graph indicates the amount of transition between each of the screens with the thickness of the lines. The same organization is used in the following figures.

The session starts with a “work as usual" phase in which each operator is in charge of a state as would be the case in the control room (*Phase 1*). After 5 min, the trainers start a cyber attack which consists of erroneous data coming from stations of a power providing company (*Phase 2*). Once the operators notice the issue, they needed to contact the power company and identify the source of the problem. P1 and P2 participated in this session. Figure 5 shows the results for the session.

#### 4.2.2. P1

There was too much noise in the ECG data from P1 for this session and we could not compute the HRV. We do not have PANAS results for P1 for this session.

*Workload Profile*

Workload was estimated rather high by P1 in the initial phase of this session (WP = 71). We can expect that in addition to monitoring their state, operators are already looking for anomalies in the data, which adds a cognitive overhead. This can be confirmed by the fact that both *Visual* and *Auditory* input processing is rated high, suggesting that P1 is processing a large amount of information, and that both *Manual* and *Speech* responses are also high, suggesting that P1 is actively looking. When the cyber-attack is discovered, P1 felt the workload decreased substantially (WP = 15).

#### 4.2.3. P2

*Workload Profile*

Similar to P1, P2's workload is high from the beginning (WP = 59), for the same reasons. When the cyber-attack is discovered, their workload increased (WP = 81).

*Heart Rate Variability*

The stress level of P2 slightly decreased between Phase 1 and Phase 2, following the workload. HRV = 0.79 at the beginning, HRV = 0.815 at the end.

*Screen Usage*

During Phase 1, P2 looked mostly at screen 5, on the application that handles the market (SOMMS—System Outlook for Market Management System: a wholesale system which is responsible for determining the cost of energy and provides functions such as ancillary services, dispatch, market information, NEM reports, settlements and prudentials and trading facilities) (≈50%). When looking at the inter-screens trajectories, we can see that P2 performed back and forth between the main application on which they focus on (on Screen 5) and the other screens, either to get information or to monitor specific parameters. In Phase 2, P2's focus is more distributed. This can be explained by the fact that P2 is in charge of the actions to resolve the issue, and thus gets information in different applications.

*PANAS*

P2 felt the session did not really affect their feelings.

#### 4.2.4. Summary

Overall, when looking at the HRV and the PANAS, the cognitive load during this scenario seemed very stable, meaning that the actions to take when the cyberattack is discovered are not stress-inducing. Surprisingly, participants rated the workload differently. For both participants, the workload was considered high in Phase 1. While Phase 1 is supposed to be work as usual, we can expect that in addition to managing their state, they are already looking for anomalies in the data, which added a cognitive overhead. In Phase 2, P1 felt the workload decreased substantially, while P2 felt it increased. The difference can be explained by the task distribution after the cyberattack is discovered.

### 4.3. Training 3: Tasmanian Bushfires (P1 and P2)

#### 4.3.1. Context

**Figure 6:**
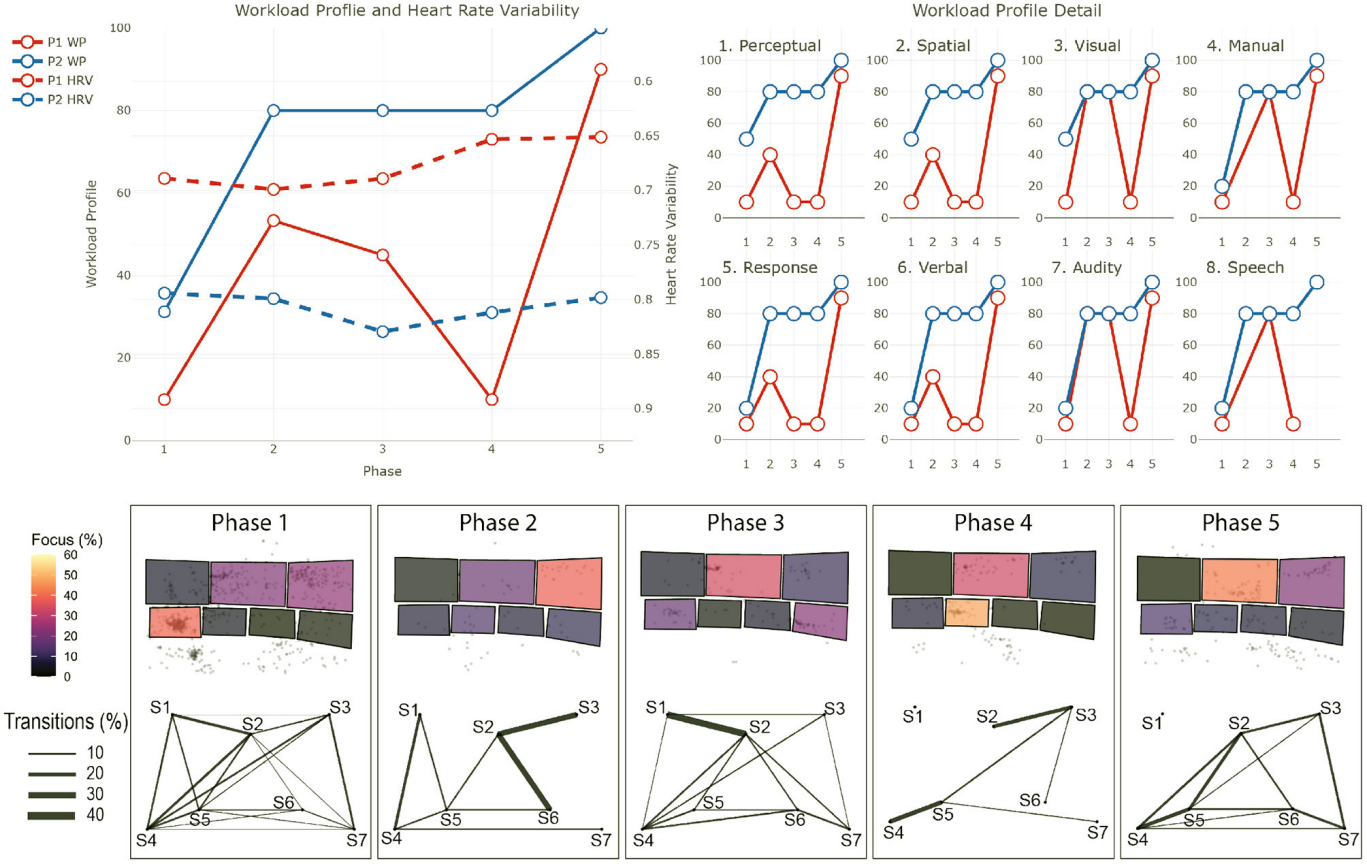
Results for the first session of training 3. The top-left chart shows the evolution of the WP and HRV during the session for both participants (the axis of the HRV is inverted). The top-right chart shows the evolution of each criteria of the WP during the session for both participants. The bottom chart displays eye tracking data for P2 for each phase: at the top is the schematic representation of the operator's workspace with a heatmap showing the amount of time the operator spent focusing on each screen; the bottom graph indicates the amount of transition between each of the screens with the thickness of the lines. The same organization is used in the following figures.

**Figure 7:**
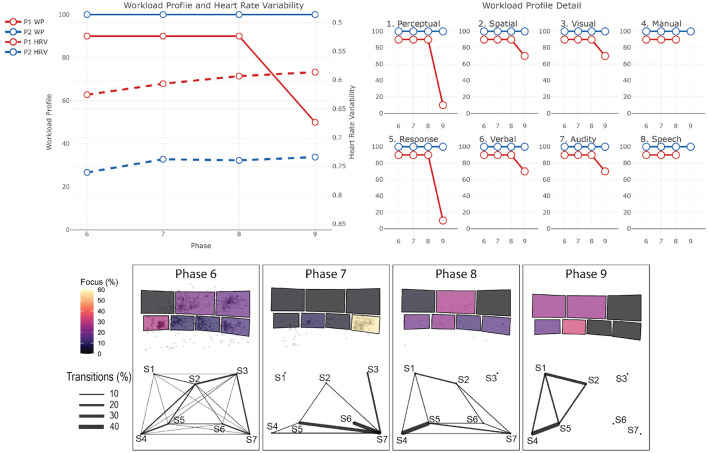
Results for the second session of training 3. The top-left chart shows the evolution of the WP and HRV during the session for both participants (the axis of the HRV is inverted). The top-right chart shows the evolution of each criteria of the WP during the session for both participants. The bottom chart displays eye tracking data for P2 for each phase: at the top is the schematic representation of the operator's workspace with a heatmap showing the amount of time the operator spent focusing on each screen; the bottom graph indicates the amount of transition between each of the screens with the thickness of the lines. The same organization is used in the following figures.

This training is divided into two sessions. In this training, the operators faced an important bushfire event in Tasmania which impacted several important lines in the network and led to the separation of the northern and southern network on the island. The scenario starts with a “Work as usual” phase, in which operators prepare their workspace and get an awareness of the current situation on the network (*Phase 1*). Soon, there are reports of fire near the lines in Tasmania (*Phase 2*). As the lines in question are the only link between the northern and southern networks, the operators have to assess the risk of separation (*Phase 3*) and configure the network accordingly (*Phase 4*). The first session ends with a first line failing because of the fire (*Phase 5*). The second session starts with the reconfiguration of the network due to the fact that only one line is now connecting the two networks (*Phase 6*). The second line fails shortly after, leading to the separation event (*Phase 7*). After managing the event and making sure that enough power is generated in both networks (*Phase 8*), the operators had to restore the lines (*Phase 9*). Figure 6 shows the results for the first session and Figure 7 shows them for the second session.

#### 4.3.2. P1 - Session 1

*Workload Profile*

P1 assessed their workload as rather low in the initial phase (WP = 10), but it increased sharply with the report of the fire (WP = 53). Both dimensions associated with the stage of processing increased, meaning that P1 needed to focus more on the situation and think on how to respond to it. This is confirmed by a sharp increase in the load associated to *Visual* and *Auditory* processing, meaning that they received a lot of information. Next, operators needed to reconfigure the network to take this risk into account. P1's workload decreased slightly (WP = 45), we can see that the actions have been decided before (low *Perceptual* and *Response* processing) and they are mainly performing tasks and communicating verbally (high *Manual* and *Speech* response). In the next phase, the operator needed to check the secure status of the network in the new configuration. This involved mostly discussion and checking of every specific part of the network. P1 seemed to be mostly observing during this phase as their workload decreased sharply (WP = 10), however, their stress level increased due to the complexity of the situation (HRV = 0.65). The session ended with one line failing, this sudden event (although anticipated) provoked a surge of workload (WP = 90), many actions had to be done quickly to mitigate the immediate risks for the network. Their stress level stayed high at this stage (HRV = 0.65). Overall, the session was positive for P1 as they felt more “Determined,” “Attentive,” and “Active” after. They also felt more “Jittery” and “Nervous,” which could indicate that the session was indeed stress inducing.

*Heart Rate Variability*

Up until the third phase, P1's stress level is almost constant (HRV ≈ 0.69). In Phase 4, even if P1's workload greatly decreased, as mostly observing, their stress level increased due to the complexity of the situation (HRV = 0.65). Their stress level stayed high at the last stage (HRV = 0.65).

*PANAS*

Overall, the session was positive for P1 as they felt more “Determined,” “Attentive,” and “Active” after. They also felt more “Jittery” and “Nervous,” which could indicate that the session was indeed stress inducing.

#### 4.3.3. P2 - Session 1

*Workload Profile*

P2 assessed their workload as low at the beginning of the session (WP = 31), with a high load for *Perceptual*, and *Spatial* processing, which could indicate that P2 was actively gathering awareness of the current situation. Workload increased sharply with the reports of fire (WP = 80) with an increase in all dimensions. Their workload stayed stable until the moment the first line failed, in which their workload maxed (WP = 100).

*Heart Rate Variability*

Regarding P2's stress level, it reduced until the reconfiguration of the network (HRV = 0.79 at the beginning, HRV = 0.83 then), then, similar to P1, stress levels increased, probably due to the complexity of the situation (HRV = 0.80 at the end).

*Screen Usage*

In Phase 1, P2 mostly gather awareness about the current situation. They spent 40% of the time on screen 4, reading and applying action from a procedure that had to be done at the beginning of the session. Most actions had to be done on screen 2 and 3, which explains frequent back and forth between screen 4 and those ones. In Phase 2, P2 spent most of this phase discussing with the other operators using the coordination screen and thus did not spend much time looking at their workstation. P2 spent most of the fourth phase doing load computation on screen 5 (over 50% of the time), with back and forth with screen 2 to get information. The line failure is signaled to operators with an audio alarm, this led to a shift of attention of P2 to the alarm summary application on screen 2, which will be mostly used until the end (around 45% of the time), with the network diagram on screen 3 (around 30% of the time), with various back and forth with the other displays to get information.

*PANAS*

Overall the session was very positive for P2 as we can see an increase in 8 out of 10 positive feelings.

#### 4.3.4. P1 - Session 2

*Workload Profile*

Session 2 started directly with the reconfiguration of the network due to one of the line failing in the end of the previous session. P1 felt directly an increased workload (WP = 90). The workload will stay stable for most of the session until decreasing for the line restoration (WP = 50). Both *Perceptual* and *Response* processing decreased sharply, which could indicate that P1 is mostly observing for this last phase.

*Heart Rate Variability*

P1's stress level increased during the entire session (HRV = 0.63 at the beginning, HRV = 0.59 at the end).

*PANAS*

P1 saw this session as very positive, with a big increase of “Attentive,” “Alert,” and “Active” feeling. They also felt much more “Jittery” and “Nervous” at the end of the session, which confirmed the stress induced by the session.

#### 4.3.5. P2 - Session 2

*Workload Profile*

Similarly to P1, P2 felt a very high workload from the beginning (WP = 100) which stayed stable the entire session. On the questionnaire, P2 actually commented that they felt their workload was so high, they could not do all the tasks that were required.

*Heart Rate Variability*

P2's stress also increased all along the session (HRV = 0.76 at the beginning, HRV = 0.73 at the end).

*Screen Usage*

When looking at the focus of P2, we can see that they spent the beginning of the session following a procedure to reconfigure the network displayed on screen 4 (around 40% of the time), this required a lot of back and forth with the other screens. When the second line failed, operators needed to reconfigure the network again. P2 spent most their time on screen 3 first (around 50% of the time) looking at network diagram and then screen 2 (around 60% of the time) looking at Contingency Violations Summary (wherein operator looks at the details of power lines and their status) and short circuit results details. During the same period, P2 also focused a large portion of the time on screen 7 (wherein constraint management and marketing management system applications are open), doing lots of back and forth between this screen and the other lower ones (screen 4, 5, and 6). Overall, the separation required the operator to use extensively up to 5 applications that required information and actions on other, sometimes distant, screens. The line restoration was mostly done on the coordination screen.

*PANAS*

P2 felt this session was rather negative as 9 out of 10 positive feelings decreased between before and after the session. 3 negative feelings also increased (“Distressed,” “Jittery,” and “Nervous”), which is a first for this participant.

#### 4.3.6. Summary

In the first session, both participants felt their workload increased when the disruptive event happened. However, the effect on their stress due to this event is different for both operators. It increased P1s stress but decreased P2s. One reason for this difference, in addition to different roles in the management of the event, could be that due to long experience as an operator, P2 has already met such a situation several times and knows how to handle it. Another explanation could be that after the bush fire report (when the second phase started), there was a vast exchange of communication and ideas between operators while they were looking for details on the coordination screen. That social environment may help give a better understanding of the disruptive event on P2s part and hence lower levels of stress. Following this event, P2's stress level stabilized despite the workload increasing. On the other hand, P1's stress increased while their workload decreased. It can be explained by a general atmosphere in the room during the simulation which could be stress-inducing as operators are facing a situation that is becoming more and more complex. The PANAS showed that even if it was a stressful session, it was overall positive for both participants, and they felt more Active, Determined and Attentive showing little signs of being cognitively overloaded.

Overall, it is fair to say that the second session was very demanding. First, the effects on the positive affect are the biggest registered in this study. Surprisingly, they are opposite for both participants. While P1 felt more positive after, it was overall less positive for P2. One reason could be a large difference in experience between the two participants. The impact of experience on cognitive and positive/negative affect should be investigated further in this context. Both WP and HRV showed high workload and high levels of stress. The workload decreased when operators had to restore the load, this part was actually done conjointly with the trainers, which makes it a little easier and more instructive.

When looking at the screen usage, we can see that operators were performing cognitively heavy tasks. At first, they followed a procedure for contingency reclassification. The inter screen trajectories confirm that P2 had to visit lots of screens to perform the procedure, and had to always go back to the procedure on Screen 4. Following this, they have to manage constraints, using an application already reported as stress-inducing and have to manage the impact of the tripped lines on the market. They also were looking for the possible Raise 6 s FCAS services (used to increase generation so to balance demand and supply and henceforth the frequency of the grid) during these phases. In both of these phases, the necessary to and fro between the distant screens could add to the workload. All of this shows that this scenario and session is the most cognitively heavy for participants. This is confirmed also from discussions with the trainers post recording, where it was mentioned that this training scenario was particularly demanding.

### 4.4. Real-Time Operation 1: Handover 1 and 2 (P1 and P3)

#### 4.4.1. Context

**Figure 8:**
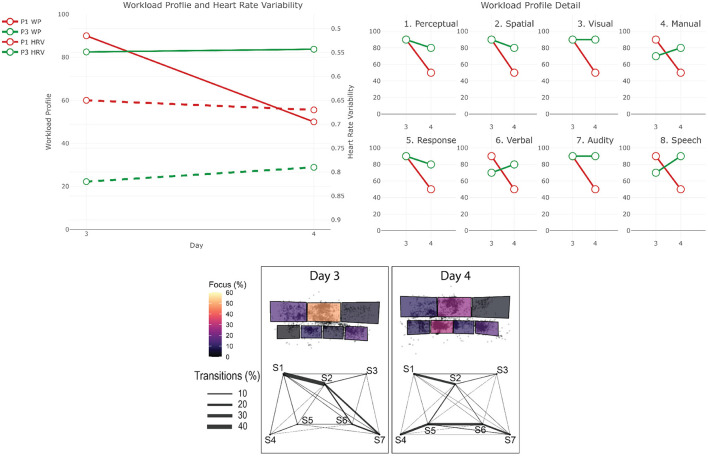
Results for the two handover sessions. The top-left chart shows the evolution of the WP and HRV during the session for both participants (the axis of the HRV is inverted). The top-right chart shows the evolution of each criteria of the WP during the session for both participants. The bottom chart displays eye tracking data for P3 for each phase: at the top is the schematic representation of the operator's workspace with a heatmap showing the amount of time the operator spent focusing on each screen; the bottom graph indicates the amount of transition between each of the screens with the thickness of the lines. The same organization is used in the following figures.

As the control room is running 24 h a day, there is a day team, which is working from 6 am to 6 pm, and then a night team that takes over until 6 am the next day. Operators do a shift in one team for 5 days, then they have 5 days off, and then do a 5-days shift in the other team. The transition between the two teams in the morning and evening is called *handover* and consists of a 5 to 10 min discussion between the operators of each team at each workstation. The operator leaving has to provide awareness of the current situation with the network and of the potential known disruptions to be expected during the shift to the arriving one. We recorded 2 handover sessions between the night and day shifts on 2 consecutive days with the same operators (leaving and arriving). On the first night, both operators were coming back from their 5-days leave. Figure 8 shows the results for the two handover sessions.

#### 4.4.2. P1

*Workload Profile*

While both handover were very similar, P1 rated their workload rather high in for the first day (WP = 90), but 40 points lower on the second day (WP = 50).

*Heart Rate Variability*

While the HRV between 2 days should be treated with caution, we can see that their stress level also seemed to be lower on the second day (HRV = 0.65 on the first day, and HRV = 0.65 on the second).

*PANAS*

When looking at the PANAS, we can see that the results are very similar between the two sessions. The positive affect increased during the session and the negative one decrease. On day 3, P1 felt more Active, Proud, and Attentive, and on day 4 felt more Active, Proud, and Alert. The negative affect decreased over the session. On both days, P2 felt less nervous.

#### 4.4.3. P3

*Workload Profile*

P3 also rated their workload high on the first handover (WP = 83), but contrary to P1, it slightly increased on the second day (WP = 84). We can see that the load on *Perceptual* and *Response* processing decreased between day 3 and day 4, the load for *Manual* and *Speech* responses increased. This could mean that they needed to process less information and make less decisions, but they had to act more. The information to process seemed to be more *Verbal* than *Spatial* on day 4.

*Heart Rate Variability*

Again treated with caution, the HRV suggests their stress level increased slightly between the two sessions (HRV = 0.82 on the first day, and HRV = 0.79 on the second).

*Screen Usage*

During the first handover session, Screen 2 was the most used screen (almost 45%). Screens 7 and 1 are also extensively used. On Screen 2 the highest intensity of focus was on the Alarm Summary. From the inter screen trajectories, we can see that most are centralized on Screen 2. With the exception of several transitions between Screens 6 and 7. On the second session, P3 focused the most on Screen 5 (around 30% of the time). Then P3 focused equally on Screen 2 and 7. P3 saccaded a lot between Screen 1 and 2 which is a pattern also observed on the first session. However, the other trajectories, are, for the most part, centered on Screen 5.

*PANAS*

On day 3, the positive affect increase over the session. P3 felt more Active, Enthusiastic, Excited, Inspired, and Strong. The negative affect decreased over the session, with P3 feeling more nervous at the end of the simulation. On the contrary, on Friday, the positive affect decreased over the session. P3 felt less Active, Enthusiastic, Excited, Interested, and Determined. On the other hand, the negative affect decreased, with P3 feeling less Nervous.

#### 4.4.4. Summary

Although we have two handover sessions, when looking at the data, they seem quite different. On Thursday, P1 and P2 have a very similar feeling regarding the positive affect and the workload. On the contrary, Fridays session has been considered as positive and with a medium workload by P1, when P2 considered it as more negative with a high workload. The difference can actually be seen in the way the screens are used. Thursdays session is mostly centered on Screen 2, while the session on Friday is centered on Screen 5. This difference could be due to the fact that the two handovers were different. Thursday was the first day back at work in the control room for both operators after some days off. Thus, both needed to gain an awareness of what had happened in the last few days and what was expected. This can explain the main focus on the Alarm Summary, and the back and forth with Screen 5 and Screen 7 to gain more information about the different events. On the other hand, on Friday, the purpose of the handover is just to get an overview of what happened over the night, which requires less effort as only a few events may have occurred. When looking at the screen usage for the Friday handover, it is actually very similar to the screen usage of the operation during the day in the control room (see next section). This could be due to the fact that the first handover of the shift cycle for an operator is more interesting than a regular one, especially for a well-experienced operator as P3.

### 4.5. Real-Time Operation 2: Normal Operation 1 and 2 (P1 and P3)

#### 4.5.1. Context

**Figure 9:**
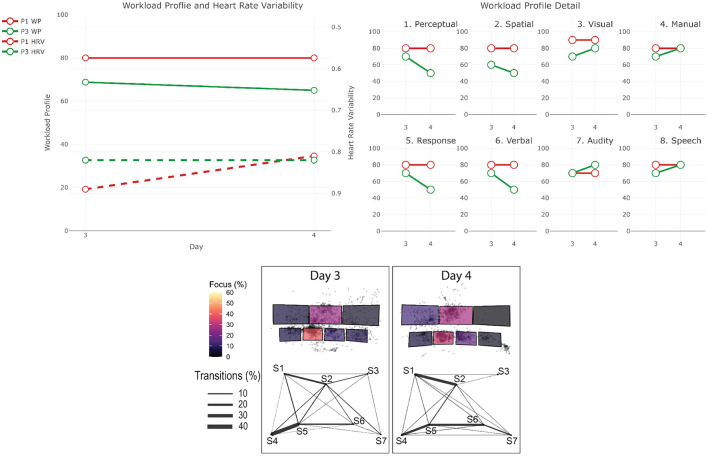
Results for the two normal operation sessions. The top-left chart shows the evolution of the WP and HRV during the session for both participants (the axis of the HRV is inverted). The top-right chart shows the evolution of each criteria of the WP during the session for both participants. The bottom chart displays eye tracking data for P3 for each phase: at the top is the schematic representation of the operator's workspace with a heatmap showing the amount of time the operator spent focusing on each screen; the bottom graph indicates the amount of transition between each of the screens with the thickness of the lines. The same organization is used in the following figures.

During normal operations (i.e., no unexpected events), operators have to monitor the network and perform planned maintenance operations. They of course have to anticipate any unexpected event. We recorded 2 normal operation sessions during 2 consecutive afternoons. No unexpected events happened during these sessions. Figure 9 shows the results for the two normal operation sessions.

#### 4.5.2. P1

We do not have the PANAS data on day 3. *Workload Profile*

P1 rated the workload on day 3 and 4 as high (WP = 80).

*Heart Rate Variability*

The HRV data suggests that the stress level increased between the two sessions (HRV = 0.89 on the first day, and HRV = 0.81 on the second).

*PANAS*

On day 4, when looking at the PANAS, we can see that the positive affect increased, while the negative stayed stable. After the session, P3 felt more Enthusiastic, Excited, and Determined.

#### 4.5.3. P3

*Workload Profile*

In general, P3 rated both sessions very similarly. They assessed their workload as a little high but stable between both sessions (WP = 68.8 on the first day and WP = 65 on the second day).

*Heart Rate Variability*

The HRV data suggests that the stress level do not really change between the 2 days (HRV = 0.82).

*Screen Usage*

Both screen usage and inter screen trajectory are very similar between the 2 days. P3 focused more on Screen 5 (around 50% of the time). During the whole scenario, the application related to the management of the market was used most of the time. The trajectories are mostly centered on Screen 5, with also a few back and forth transitions between Screen 1 and 2 and between Screens 7 and 2 on day 3.

*PANAS*

We can see that the evolution of the positive affect is smaller than during the handover sessions. Positive and negative affect did not increase during the session on day 3. The positive one slightly increases on day 4, with P3 feeling more Excited and Alert, but less Strong. The negative affect of P3 also decreased, P3 felt less Nervous.

#### 4.5.4. Summary

Overall, the data we collected show two similar sessions. While P3 felt the workload was a little high, it is not the case of P1. This could be due to the difference of role and experience. The session did not negatively impact the affect of the participants. Regarding the use of screen during normal operation. We can see a main focus on screen 5 and then lots of back and forth with other screens, probably to look for information or act on specific parameters.

## 5. Discussion

Through the results of this study, we have highlighted a number of interesting insights into the individual operators cognitive load during both the training and control room scenarios. Relationships between cognitive load and design aspects of the system are discussed.

### 5.1. Operators, Tasks and Applications

In this study, by assessing the variation of both WP and HRV, we can see that cognitive load varies in the different training scenarios and control room operations. This change of cognitive load is mostly caused by differences in the situation and in the operators actions. For instance, if we look at the Handover, we can see that P3's WP is quite high, but is substantially lower in Normal Operation. This difference can be easily explained by both the different situations, and thus, the different tasks the operator has to do. However, it is inferred that there are also situations where excess cognitive load is induced by inefficient system design—particularly, aspects of the system that are optimized for some tasks adversely affect other tasks. This is a good demonstration of the necessity to measure cognitive load in a variety of situations instead of focusing on only one. On the other hand, this necessitates a more observational rather than controlled study design and hence broader results.

In addition to differences between tasks, our results suggest that cognitive load is different between operators for the same task. A good example of such situation is the first session of Training 3. When the first fire is reported and the operator had to assess a possible separation, P1's stress level increase (HRV decrease), but P2's one decrease (HRV increase). Operators have varying experience, which influences how they deal with a situation. This phenomenon has been formalized by Rasmussen with the SRK (Skills, Rules, Knowledge) model which defines the different levels of decision making and how training and experience can influence them (Rasmussen, 1983). Further research using this model would be necessary to better understand the impact that experience has on cognitive load. Another reason could be that the role of operators is different for a given situation.

Interestingly, in our study, we notice several differences between how operators handle the situation in the control room and in the training scenario. We observed that work in the control room is often centered and focused on one screen for long periods (In screen 2 for the handover for instance). When needing additional information, or for short tasks, the operator focuses on other screens. In training, the situations are generally much more complex, and thus the operators need to spread their focus over multiple screens (A good example can be the second phase of Training 2). Even in a phase where operators are supposed to ‘work as usual’ in training, their process was completely different as they were looking for something unusual.

The two handover sessions we observed were quite different in how the operator handles them and how it impacted their mental state. In the first, the operator was trying to get situational awareness of what had happened in the last 4–5 days, since the last shift rotation. Their focus was thus spread across multiple screens compared to the second handover session where they only had to understand what had occurred during the night. The activities during the second handover session are very similar to those during normal control room operation. While the first handover was more cognitively demanding, it was also more interesting and satisfying for the operators.

Our analysis of the inter-screen visual trajectories in general suggests that operators had to frequently shift their attention between applications located on different screens, leading to an increase in cognitive load. Session 2 of Training 3 offers a good example of such behavior with P2 following a procedure for contingency reclassification and thus having to visit several screens to get information and act on elements from the network. In addition to the time to travel from one screen to another, it will take more time for an operator to locate the information after a long visual path than a short one. Such effects have been verified for visual comparison by Plumlee and Ware (2006). In addition, the screen glance is often very quick, meaning that operators often need to go to the other application only to find information linked directly to the task at hand.

Our results, observations and discussions with operators, suggest that some applications lead to more stress and cognitive load (e.g., one application, the constraint manager, requires the operator to fill a lot of information and is not very ergonomic. It was extensively used by P2 in the Training 1 Session 2.). This seems to be particularly labor intensive to use, and can be slow or unresponsive, when needing to urgently enter market constraints. The lack of responsiveness, small font size and lack of confirmation pop-ups make it susceptible to data entry mistakes so extra attention when using the interface is needed.

### 5.2. Collaboration, Procedures and Alarms

The management of a critical situation can be relatively collaborative. To deal with an unusual event, the operators will extensively discuss the different alternatives. Workload could be high but there may not be an increase in cognitive load of operators because they are working collaboratively together, hence providing social support. Thus, a task could be hard for an operator but working together takes away the stress of not having all the answers. Our study was not designed to analyse the collaborative aspect of these situations. For this first study, it was necessary to better understand the impact at an individual level. Further research exploring operator collaboration would be necessary to get a full picture of the situation.

Due to the critical nature of the tasks of the operators, they have to follow, for most of their actions, very strict and detailed procedures. These procedures are documents that operators need to manually search for and open, depending on the situation at hand. They are not linked to other applications within the system. When following a procedure, an operator will have it open on one screen, and will look back and forth between the procedure and other screens in order to perform the different actions. Following the procedure was sometimes a collaborative process, where one operator was reading it aloud while the others carried out the actions. This is partly what was done in the second session of Training 3, in which P2 followed the procedure for contingency reclassification.

The training scenarios in particular saw extensive collaboration between the operators in the same room, and between the two rooms. In these instances, the coordination screen was often used to share documents, the procedure, or display the video stream of the other center. A good example is the first session of the Training 3, with the report of the fire, the operators used the coordination to debate and decide if there is a risk of separation. This screen is however not fully integrated into the main system.

An important part of the conversation between the two centers is about what an operator is doing. Workspace awareness is a concept defined by Dourish and Bellotti (1992) as the understanding of the activity of others in order to give a context to one's own activity. In the training scenario, we observed that this workspace awareness was mostly shared by having the operators describing orally their actions, and that it represents a high percentage of the information transmitted during the conversion between the two centers.

Our observations and discussions also highlighted that each alarm in the system has a unique sound and that these different sounds had been chosen and added relatively randomly over time. Operators learn these through training and practice. The sheer number and intensity of alarms can pose a problem for operators' recall and can increase cognitive load. Prioritizing these quickly on demand is difficult in many control room environments (Momtahan et al., 1993). In both the control and training room scenarios certain sounds are more evident in their pitch, sound wave and amplitude than others.

## 6. Recommendations

With these insights we identify some recommendations for possible implementation or continued research:

### 6.1. Optimize Operator Application Arrangements

At the start of each shift, operators spend time arranging their layout and throughout the day spend time looking at different applications across different locations on their workstation. Whilst applications are arranged by operators based on the perceived optimal layout of their workload, it is clear from the eye-tracking data from a number of the sessions, that this is not always optimal for the task at hand. Further work to improve application arrangement and therefore reduce visual distance between applications frequently used could include:

**Arrangement Strategies**: a study of operator application arrangements could help to understand different strategies for operators at these advanced workstations and identify common patterns with application position as well as its proximity and association to others.

**Profile loading**: an automated profile loading script for operators could be developed to load their preferred profile and arrange their applications in a set manner and preferences. This can be based on their personal preferences, but also using strategic arrangements based on the study of operators. Operators might even want to switch profile type mid-shift, when working on a particular task.

**Optimal Arrangements**: further exploration of which applications are typically used in conjunction with each other for certain tasks could help design optimal application arrangement profiles.

### 6.2. Linked Views, Associations and Performance

Long fixations on single applications during some sessions, phases or tasks indicate that some applications are used more often or for longer periods than others. Movement between pairs of applications is also prevalent. Therefore, we recommend:

**Combining applications**: Explore combining key applications where information is complementary. For example overlaying the network with the region/weather information, could improve the efficiency of situation awareness for a region at the start of the shift.

**Linked Views**: Another way to facilitate the transition between two applications with linked information is to visually link the information. Information visualization research extensively explore this aspect, but very few studies focused on such a large scale regarding the number of different views/applications. It would be interesting to try different alternatives and study their impact. Colors, but also visual links could be used. In addition, it would be possible to link a non visual element (e.g., an alarm), to the relevant application. For instance, when an alarm is sounding for a particular reason an alarm icon can be seen in the diagram.

**Explore the redesign of certain applications**: SOMMS, for instance, seems to often be unresponsive, takes time to enter information and has a very small font. User experience (UX) design could improve the efficiency of the task and also the ability to multitask.

### 6.3. Coordination Screens and Documents

The procedure document was a key area of focus for training scenarios. Whilst the procedure is obviously vital to follow in these scenarios, with little development the current static digital document could be more tightly integrated into the operations, as follows:

**Collaborative and Interactive Procedures**: The procedure could be designed as a shared interactive decision tree to help follow the procedure, which updates as decisions are taken. This could be helpful in gaining a quick overview of a scenario and help to see the key decisions necessary going forward. A digitally shared procedure would not only improve the efficiency of the operators individual workflow, but collaboration between sites would be aided through a tool, which allows both sites to see who is responsible for what and who has done what. This may not only help to document processes but could also provide new simulation training for future based on real scenarios.

**Coordinated Screen or pointers**: the ability to quickly be able to see a colleague's screen or their mouse location, could help you gain quick insight of what other operators are working on during an emergency situation via a visual cue. This could allow operators to shared workspace awareness without extensive conversation and thus allow them to focus more on the problem at hand.

**Better integration of the Coordination Screen**: The coordination screen is extensively used when collaborating, but it is not fully integrated in the system. It would be possible to design this screen for inter-center collaboration and allow operators to display several application windows from both centers simultaneously.

### 6.4. Optimize Alarm Prioritization and Improve Association

Alarm sounds were present throughout all scenarios with certain sounds being more evident than others. The operators' association of these alarms to the situation at hand could be improved through a number of options, including:

**Visual cues to highlight relevant applications**: through linking the backend of the systems, selected critical alarms could be linked to relevant applications associated with the alarm or a particular region in the diagram for instance. Such addition visual cue can aide operators in quickly identifying, associating and prioritizing these alarms quickly.

**Assess alarm sounds**: a controlled study of the control room alarms using type and pitch changes would help ensure that the sounds are appropriately associated with urgency and thus stress levels are not unnecessarily overly increased or decreased.

## 7. Conclusion

Cognitive load refers to the amount of mental resources required to perform and learn from a task. In this research article we studied the cognitive load of operators in an energy network control room during normal control room operations and unexpected events simulated in training scenarios (extreme weather events, cyber attacks, and IT failure). An integrated approach of using both subjective and objective measures was applied to evaluate cognitive load of operators.

Our results allowed us to identify several factors in the design of the control system that could increase the cognitive load of operator. These include the layout of different applications, the lack of integration of the procedures in the system and the limited support for intense coordination between the operators during unexpected events. We proposed a set of recommendations for the design of future systems that would mitigate the effect of the identified factors. It would be interesting to implement the recommendations and to assess their impact. Further studies are needed to investigate the impact of some of our insights, such as the degree to which operator collaboration or a different set of alarms can impact cognitive load. These recommendations are likely to also be applicable to other control room environments where operators are required to maintain a high level of situational awareness, such as in nuclear power plant control rooms, air traffic control rooms, control rooms in process industry. We encourage further research in these areas.

## Data Availability Statement

The original contributions presented in the study are included in the article/Supplementary Material, further inquiries can be directed to the corresponding author.

## Ethics Statement

The studies involving human participants were reviewed and approved by Monash University Human Research Ethics Committee (MUHREC). The patients/participants provided their written informed consent to participate in this study.

## Author Contributions

SG, AP, LL, AL, and TD conceived the idea and design of the experimentation. SG, AP, LL, UA, and SB collected the data. SG, AP, LL, and UA pre-processed, processed, analyzed, and interpreted the data. UA, AP, and SG wrote the manuscript. LL, TD, and AL reviewed the article and helped formulating the last version of the manuscript. All authors approved the final version of the article and contributed to the article.

## Funding

This study was funded and supported by Australian Energy market Operator (AEMO).

## Conflict of Interest

The authors declare that the research was conducted in the absence of any commercial or financial relationships that could be construed as a potential conflict of interest.

## Publisher's Note

All claims expressed in this article are solely those of the authors and do not necessarily represent those of their affiliated organizations, or those of the publisher, the editors and the reviewers. Any product that may be evaluated in this article, or claim that may be made by its manufacturer, is not guaranteed or endorsed by the publisher.

## Footnotes

^1^ https://shimmersensing.com/product/shimmer3-ecg-unit-2/

^2^ https://www.tobiipro.com/product-listing/tobii-pro-glasses-2/

## References

[B1] Astic J.-Y. Bareux G. Buhagiar T. Dussartre M. Omont N. de Longeaux P.. (2018). Control center designs: new functions and challenges for the transmission system operator. IEEE Power Energy Mag. 16, 57–66. 10.1109/MPE.2017.2779553

[B2] Bhavsar P. Srinivasan B. Srinivasan R. (2017). Quantifying situation awareness of control room operators using eye-gaze behavior. Comput. Chem. Eng. 106, 191–201. 10.1016/j.compchemeng.2017.06.004

[B3] Brouwers S. Wiggins M. W. Helton W. OHare D. Griffin B. (2016). Cue utilization and cognitive load in novel task performance. Front. Psychol. 7, 435. 10.3389/fpsyg.2016.00435 27064669 PMC4809880

[B4] Dadashi N. Wilson J. R. Golightly D. Sharples S. (2016). Alarm handling for health monitoring: operator strategies used in an electrical control room of a rail network. Proc. Institut. Mech. Eng. F J. Rail Rapid Transit 230, 1415–1428. 10.1177/0954409715593574

[B5] Dan A. Reiner M. (2017). Real time eeg based measurements of cognitive load indicates mental states during learning. J. Educ. Data Min. 9, 31–44. 10.5281/zenodo.3554719

[B6] Dehais F. Causse M. Vachon F. Régis N. Menant E. Tremblay S. (2014). Failure to detect critical auditory alerts in the cockpit: evidence for inattentional deafness. Hum. Factors 56, 631–644. 10.1177/0018720813510735 25029890

[B7] Dourish P. Bellotti V. (1992). Awareness and coordination in shared workspaces, in Proceedings of the 1992 ACM Conference on Computer-Supported Cooperative Work (Cambridge, UK: ACM CSCW), 107–114.

[B8] Eggemeier F. T. Wilson G. F. Kramer A. F. Damos D. L. (1991). Workload assessment in multi-task environments, in Multiple-Task Performance (London, UK), 207–216.

[B9] Endsley M. R. (1995). Toward a theory of situation awareness in dynamic systems. Hum. Factors J. 37, 32–64. 10.1518/001872095779049543

[B10] Fallahi M. Motamedzade M. Heidarimoghadam R. Soltanian A. R. Miyake S. (2016a). Assessment of operators mental workload using physiological and subjective measures in cement, city traffic and power plant control centers. Health Promot. Perspect. 6, 96. 10.15171/hpp.2016.17 27386425 PMC4932229

[B11] Fallahi M. Motamedzade M. Heidarimoghadam R. Soltanian A. R. Miyake S. (2016b). Effects of mental workload on physiological and subjective responses during traffic density monitoring: a field study. Appl. Ergon. 52, 95–103. 10.1016/j.apergo.2015.07.009 26360199

[B12] Ghanbary Sartang A. Ashnagar M. Habibi E. Sadeghi S. (2016). Evaluation of rating scale mental effort (rsme) effectiveness for mental workload assessment in nurses. J. Occupat. Health Epidemiol. 5, 211–217. 10.18869/acadpub.johe.5.4.211

[B13] Giraudet L. Berenger M. Imbert J.-P. Tremblay S. Causse M. (2014). Inattentional deafness in simulated air traffic control tasks: A behavioral and p300 analysis, in 5th International Conference on Applied Human Factors and Ergonomics (AHFE 2014) (Krakow, PL), 1–10.

[B14] Giri J. Parashar M. Trehern J. Madani V. (2012). The Situation room: control center analytics for enhanced situational awareness. IEEE Power Energy Mag. 10, 24–39. 10.1109/MPE.2012.2205316

[B15] Hancock G. Longo L. Young M. Hancock P. (2021). Mental workload, in Handbook of Human Factors and Ergonomics (New York, NY), 203–226.

[B16] Hollnagel E. Woods D. D. (2005). Joint Cognitive Systems: Foundations of Cognitive Systems Engineering. Boca Raton, FL: CRC Press.

[B17] Kim D. Seo Y. Cho J. Cho C.-H. (2008). Detection of subjects with higher self-reporting stress scores using heart rate variability patterns during the day, in 2008 30th Annual International Conference of the IEEE Engineering in Medicine and Biology Society, (Vancouver, BC: IEEE), 682–685. 10.1109/IEMBS.2008.4649244 19162747

[B18] Krejtz K. Duchowski A. T. Niedzielska A. Biele C. Krejtz I. (2018). Eye tracking cognitive load using pupil diameter and microsaccades with fixed gaze. PLoS ONE 13, e0203629. 10.1371/journal.pone.0203629 30216385 PMC6138399

[B19] Liu Y.-B. Liu J.-Y. Taylor G. Liu T.-J. Gou J. Zhang X. (2016). Situational awareness architecture for smart grids developed in accordance with dispatchers thought process: a review. Front. Inf. Technol. Electron. Eng. 17, 1107–1121. 10.1631/FITEE.1601516

[B20] Longo L. (2018). On the reliability, validity and sensitivity of three mental workload assessment techniques for the evaluation of instructional designs: a case study in a third-level course, in CSEDU (2), The Global Centre of Excellence for Digital Content and Media Innovation, Dublin, Ireland. CSEDU 2018 - 10th International Conference on Computer Supported Education (Funchal), 166–178.

[B21] Malik M. Camm A. J. (1990). Heart rate variability. Clin. Cardiol. 13, 570–576. 10.1002/clc.4960130811 2204508

[B22] Miller S. (2001). Workload Measures. National Advanced Driving Simulator. Iowa City, IA: University of Iowa Press.

[B23] Miyake S. (2001). Multivariate workload evaluation combining physiological and subjective measures. Int. J. Psychophysiol. 40, 233–238. 10.1016/S0167-8760(00)00191-4 11228350

[B24] Momtahan K. Hetu R. Tansley B. (1993). Audibility and identification of auditory alarms in the operating room and intensive care unit. Ergonomics 36, 1159–1176. 10.1080/00140139308967986 8223408

[B25] Muir A. Lopatto J. (2004). Final report on the August 14, 2003 blackout in the United States and Canada: causes and recommendations. US-Canada power system outage task force, Washington, DC and Ottawa, Canada. Available online at: https://www.energy.gov/sites/default/files/oeprod/DocumentsandMedia/BlackoutFinal-Web.pdf

[B26] Mumaw R. J. Roth E. M. Vicente K. J. Burns C. M. (2000). There is more to monitoring a nuclear power plant than meets the eye. Hum. Factors 42, 36–55. 10.1518/001872000779656651 10917145

[B27] Myrtek M. Deutschmann-Janicke E. Strohmaier H. Zimmermann W. Lawerenz S. Brügner G.. (1994). Physical, mental, emotional, and subjective workload components in train drivers. Ergonomics 37, 1195–1203. 10.1080/00140139408964897 8050404

[B28] Naismith L. M. Cheung J. J. Sibbald M. Tavares W. Cavalcanti R. B. Haji F. A.. (2019). Chapter 10-using cognitive load theory to optimize simulation design, in Clinical Simulation, 2nd Edn, ed Chiniara G. (Cambridge, MA: Academic Press), 129–141.

[B29] NASA (1986). NASA Task Load Index (TLX) v. 1.0 Manual. Technical report, National Aeronautics and Space Agency. Available online at: http://humansystems.arc.nasa.gov/groups/TLX/downloads/TLX.pdf.

[B30] Orru G. Longo L. (2019). The evolution of cognitive load theory and the measurement of its intrinsic, extraneous and germane loads: a review, in International Symposium on Human Mental Workload: Models and Applications (Berlin: Springer), 23–48.

[B31] Paas F. Van Merrienboer J. J. G. Adam J. (1994). Measurement of cognitive load in instructional research. Percept. Mot. Skills 79, 419–430. 10.2466/pms.1994.79.1.419 7808878

[B32] Parkes A. Coleman N. (1990). Route guidance systems: a comparison of methods of presenting directional information to the driver, in Contemporary Ergonomics (London, UK), 480–485.

[B33] Pawar S. Jacques T. Deshpande K. Pusapati R. Meguerdichian M. J. (2018). Evaluation of cognitive load and emotional states during multidisciplinary critical care simulation sessions. BMJ Simulat. Technol. Enhanced Learn. 4, 87–91. 10.1136/bmjstel-2017-000225 29670763 PMC5890622

[B34] Plumlee M. D. Ware C. (2006). Zooming versus multiple window interfaces: cognitive costs of visual comparisons, in ACM Transactions on Computer-Human Interaction (TOCHI) (New York, NY), 179–209.

[B35] Prouzeau A. (2017). Collaboration around wall displays in command and control contexts-Chapter 3 (Ph.D. thesis).

[B36] Qin Z. Li M. Huang L. Zhao Y. (2017). Stress level evaluation using bp neural network based on time-frequency analysis of hrv, in 2017 IEEE International Conference on Mechatronics and Automation (ICMA) (Takamatsu: IEEE), 1798–1803.

[B37] Rao H. M. Smalt C. J. Rodriguez A. Wright H. M. Mehta D. D. Brattain L. J.. (2020). Predicting cognitive load and operational performance in a simulated marksmanship task. Front. Hum. Neurosci. 14, 222. 10.3389/fnhum.2020.00222 32719593 PMC7350508

[B38] Rasmussen J. (1983). Skills, rules, and knowledge; signals, signs, and symbols, and other distinctions in human performance models. IEEE Trans. Systems Man Cybernet. SMC-13, 257–266. 10.1109/TSMC.1983.6313160

[B39] Rubio S. Díaz E. Martín J. Puente J. M. (2004). Evaluation of subjective mental workload: a comparison of swat, nasa-tlx, and workload profile methods. Appl. Psychol. 53, 61–86. 10.1111/j.1464-0597.2004.00161.x

[B40] Shaffer F. Ginsberg J. P. (2017). An overview of heart rate variability metrics and norms. Front. Public Health 5:258. 10.3389/fpubh.2017.00258 29034226 PMC5624990

[B41] Sturman D. Wiggins M. W. Auton J. C. Loft S. Helton W. S. Westbrook J. I.. (2019). Control room operators cue utilization predicts cognitive resource consumption during regular operational tasks. Front. Psychol. 10, 1967. 10.3389/fpsyg.2019.01967 31507501 PMC6718724

[B42] Sweller J. (1988). Cognitive load during problem solving: effects on learning. Cogn. Sci. 12, 257–285. 10.1207/s15516709cog1202_4

[B43] Sweller J. Van Merrienboer J. J. Paas F. G. (1998). Cognitive architecture and instructional design. Educ. Psychol. Rev. 10, 251–296. 10.1023/A:1022193728205

[B44] Tao D. Tan H. Wang H. Zhang X. Qu X. Zhang T. (2019). A systematic review of physiological measures of mental workload. Int. J. Environ. Res. Public Health 16, 2716. 10.3390/ijerph16152716 31366058 PMC6696017

[B45] Tsang P. S. Velazquez V. L. (1996). Diagnosticity and multidimensional subjective workload ratings. Ergonomics 39, 358–381. 10.1080/00140139608964470 8849491

[B46] Tsang P. S. Vidulich M. A. (2006). Mental workload and situation awareness, in Handbook of Human Factors and Ergonomics (John Wiley & Sons, Ltd.,), 243–268. Available online at: https://onlinelibrary.wiley.com/doi/abs/10.1002/0470048204.ch9 10.1002/0470048204.ch9

[B47] Tukey J. W. (1977). Exploratory Data Analysis. Reading, MA: Addison-Wesley.

[B48] Watson D. Clark L. A. Tellegen A. (1988). Development and validation of brief measures of positive and negative affect: the panas scales. J. Pers. Soc. Psychol. 54, 1063. 10.1037/0022-3514.54.6.1063 3397865

[B49] Wicherts J. M. Veldkamp C. L. S. Augusteijn H. E. M. Bakker M. van Aert R. C. M. van Assen M. A. L. M. (2016). Degrees of freedom in planning, running, analyzing, and reporting psychological studies: a checklist to avoid p-hacking. Front. Psychol. 7, 1832. 10.3389/fpsyg.2016.01832 27933012 PMC5122713

[B50] Wickens C. D. (1980). The structure of attentional resources. Attent. Perform. VIII 8, 239–257.

[B51] Wickens C. D. (2008). Multiple resources and mental workload. Hum. Factors 50, 449–455. 10.1518/001872008X288394 18689052

[B52] Wulvik A. S. Dybvik H. Steinert M. (2020). Investigating the relationship between mental state (workload and affect) and physiology in a control room setting (ship bridge simulator). Cognit. Technol. Work 22, 95–108. 10.1007/s10111-019-00553-8

[B53] Young M. S. Brookhuis K. A. Wickens C. D. Hancock P. A. (2015). State of science: mental workload in ergonomics. Ergonomics 58, 1–17. 10.1080/00140139.2014.956151 25442818

[B54] Zagermann J. Pfeil U. Reiterer H. (2016). Measuring cognitive load using eye tracking technology in visual computing, in Proceedings of the Sixth Workshop on Beyond Time and Errors on Novel Evaluation Methods for Visualization (ACM), 78–85.

