# Supplementary material for: Investigating Cognitive Load in Energy Network Control Rooms: Recommendations for Future Designs
Source: Front Psychol. 2022 Mar 28;13:812677. doi: 10.3389/fpsyg.2022.812677 (PMC8995508; doi:10.3389/fpsyg.2022.812677)
Supplement: Supplementary file 1 [file Data_Sheet_1.PDF]

frontiers

Supplementary Material

1 Questionnaires used in the study

We used three questionnaires in this study:

1. PANAS: Directly extracted from Watson, D., Clark, L. A., & Tellegen, A. (1988).
2. Workload Profile: Directly extracted from Tsang P. S. & Velazquez V. L. (1996).
3. Demographic Questionnaire: Designed by the authors

Positive and Negative Affect Schedule (PANAS-SF)

Indicate the extent you have felt this way.		**Very slightly or not at all**	**A little**	**Moderately**	**Quite a bit**	**Extremely**
PANAS 1	Interested	☐ 1	☐ 2	☐ 3	☐ 4	☐ 5
PANAS 2	Distressed	☐ 1	☐ 2	☐ 3	☐ 4	☐ 5
PANAS 3	Excited	☐ 1	☐ 2	☐ 3	☐ 4	☐ 5
PANAS 4	Upset	☐ 1	☐ 2	☐ 3	☐ 4	☐ 5
PANAS 5	Strong	☐ 1	☐ 2	☐ 3	☐ 4	☐ 5
PANAS 6	Guilty	☐ 1	☐ 2	☐ 3	☐ 4	☐ 5
PANAS 7	Scared	☐ 1	☐ 2	☐ 3	☐ 4	☐ 5
PANAS 8	Hostile	☐ 1	☐ 2	☐ 3	☐ 4	☐ 5
PANAS 9	Enthusiastic	☐ 1	☐ 2	☐ 3	☐ 4	☐ 5
PANAS 10	Proud	☐ 1	☐ 2	☐ 3	☐ 4	☐ 5
PANAS 11	Irritable	☐ 1	☐ 2	☐ 3	☐ 4	☐ 5
PANAS 12	Alert	☐ 1	☐ 2	☐ 3	☐ 4	☐ 5
PANAS 13	Ashamed	☐ 1	☐ 2	☐ 3	☐ 4	☐ 5
PANAS 14	Inspired	☐ 1	☐ 2	☐ 3	☐ 4	☐ 5
PANAS 15	Nervous	☐ 1	☐ 2	☐ 3	☐ 4	☐ 5
PANAS 16	Determined	☐ 1	☐ 2	☐ 3	☐ 4	☐ 5
PANAS 17	Attentive	☐ 1	☐ 2	☐ 3	☐ 4	☐ 5
PANAS 18	Jittery	☐ 1	☐ 2	☐ 3	☐ 4	☐ 5
PANAS 19	Active	☐ 1	☐ 2	☐ 3	☐ 4	☐ 5
PANAS 20	Afraid	☐ 1	☐ 2	☐ 3	☐ 4	☐ 5

MONASH University

Scoring:

Positive Affect Score**:** Add the scores on items 1, 3, 5, 9, 10, 12, 14, 16, 17, and 19. Scores can range from 10 – 50, with higher scores representing higher levels of positive affect.

Negative Affect Score**:** Add the scores on items 2, 4, 6, 7, 8, 11, 13, 15, 18, and 20. Scores can range from 10 – 50, with lower scores representing lower levels of negative affect.

Your scores on the PANAS: Positive: ____ Negative: ____

Watson, D., Clark, L. A., & Tellegen, A. (1988). Development and validation of brief measures of positive and negative affect: the PANAS scales. *Journal of personality and social psychology*, *54*(6), 1063.

Workload Profile: Instructions to the participants

1) Please read the definitions of each dimension of the questionnaire. The definitions are provided along with the questionnaire.
2) After understanding the definitions, for each phase of the session (pre-filled by the experimenter, each phase is on one row) please fill the cell with a score representing how much effort in each particular dimension was required. To do so, please use a 0 to 100 value where 0 being lowest and 100 being the highest score.
3) Please note that if you feel that one dimension was not involved in a phase at all, you should not fill the cell.

	**Stage of processing**		**Processing codes**		**Input modality**		**Output modalities**	
**Task**	Perceptual processing	Response processing	Spatial processing	Verbal processing	Visual processing	Auditory processing	Manual responses	Speech responses

This sheet describe the different dimensions in order to fill the Workload Profile table. It was given to the participants before the session and explained to them if necessary.

1. **Stages of processing**
    a. **Perceptual processing**. These are the attentional resources required for activities like perceiving (detecting, recognizing, and identifying objects), remembering, problem-solving, and decision making.
    b. **Response processing**. These are attentional resources required for response selection and execution. For example, there are three foot pedals in a standard shift automobile ; to stop the automobile, we have to select the appropriate pedal and step on it.
2. **Processing codes**
    a. **Spatial processing**. Some tasks are spatial in nature. Driving, for example, requires paying attention to the position of the car, the distance between the current position of the car and the next stop sign, the geographical direction that the car is heading, etc.
    b. **Verbal processing**. Other tasks are verbal in nature. For example, reading involves primarily processing of verbal, linguistic materials.
3. **Input modality**
    a. **Visual processing**. Some tasks are performed based on the visual information received. For example, playing basketball requires visual monitoring of the physical location and velocity of the ball. Watching TV is another example of a task that requires visual resources.
    b. **Auditory processing**. Other tasks are performed based on auditory information. For example, listening to the person on the other end of the telephone is a task that requires auditory attention. Listening to music is another example.

Note that spatial information may be processed visually or auditorily. For example, you can get to a new restaurant by following a map (visual processing) or by following the direction spoken by your friend (auditory processing). Similarly, verbal information may be processed visually or auditorily. Listening to the news on the radio requires auditory processing or verbal materials ; reading the news from the newspaper requires visual processing of verbal materials.

4. **Output modalities**
    a. **Manual responses**. Some tasks require considerable attention for producing the manual response as in typing or playing a piano.
    b. **Speech responses**. Other tasks require speech responses instead. For example, engaging in a conversation requires attention for producing the speech responses.

Demographic Questionnaire

ID Participant:

Age Range:

- ❏ < 20
- ❏ 20-29
- ❏ 30-39
- ❏ 40-49
- ❏ 50-59
- ❏ 60 +

Gender:

Number of years of experience as an operator at AEMO (est.):

Number of years of experience in a similar position (est.):

Number of years of experience in the energy sector (est.):

Number of caffeinated drinks today, strength/dosage, and when:

If this recording is in the training room, how many training sessions have you done this year:
